# Supplementary material for: Imaging with spatio-temporal modelling to characterize the dynamics of plant-pathogen lesions
Source: PLoS Comput Biol. 2023 Nov 20;19(11):e1011627. doi: 10.1371/journal.pcbi.1011627 (PMC10695395; doi:10.1371/journal.pcbi.1011627)
Supplement: S1 Appendix — (PDF) [file pcbi.1011627.s001.pdf]

Imaging with spatio-temporal modelling to characterize the dynamics of plant-pathogen lesions
Appendix S1

Melen Leclerc[1], Stéphane Jumel[1], Frédéric M. Hamelin[1], Rémi Treilhaud[1], Nicolas Parisey[1], and Youcef Mammeri[2]

[1]IGEPP, INRAE, Institut Agro, University of Rennes, Rennes, France
[2]ICJ, CNRS, Jean Monnet University, Saint-Etienne, France

S1 Experimental protocol

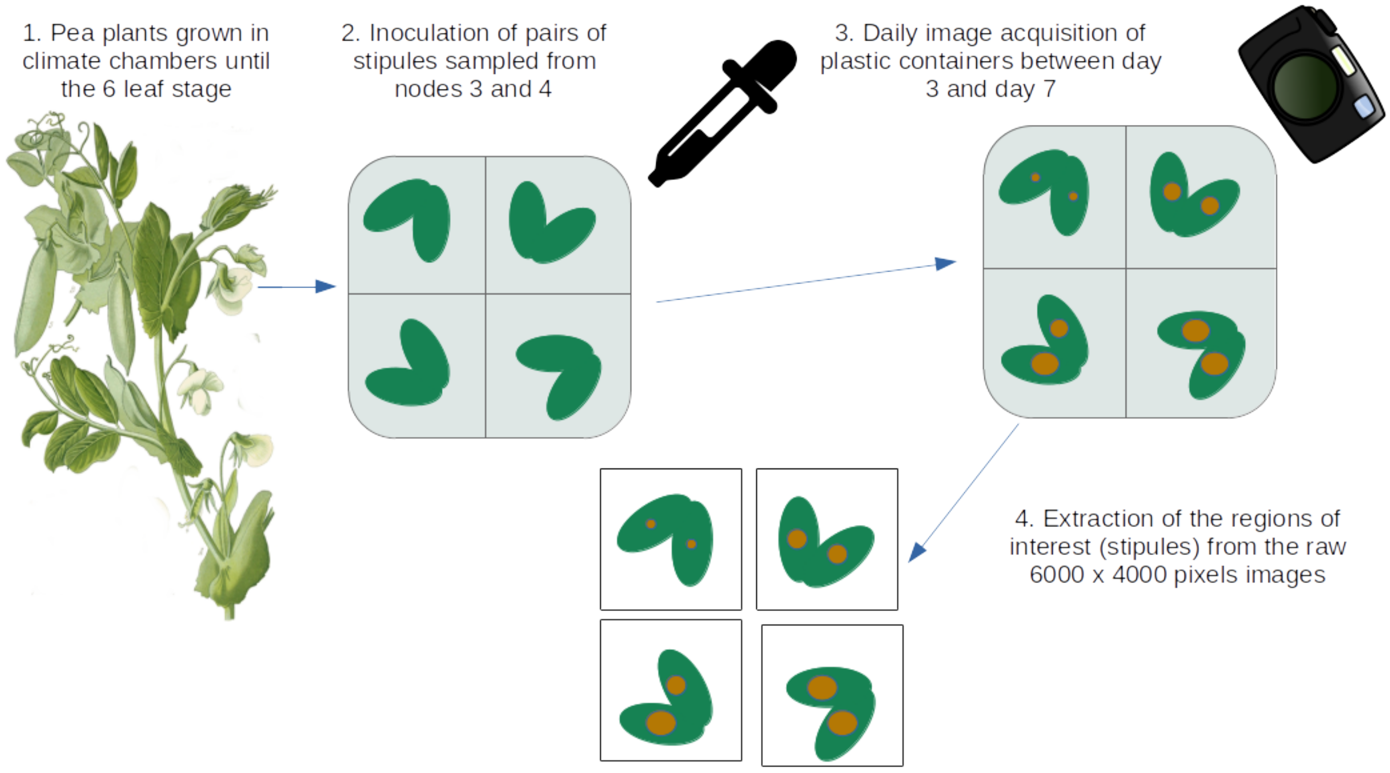

Figure A: Schematic representation of the experimental protocol. The figure was made in LibreOffice impress using open-source cliparts (https://openclipart.org/detail/190961/color-picker for the pipette, https://openclipart.org/detail/203965/camera-digital for the camera) and figure (https://www.biodiversitylibrary.org/page/4321350 for the pea plant).
