# Supplementary material for: Imaging with spatio-temporal modelling to characterize the dynamics of plant-pathogen lesions
Source: PLoS Comput Biol. 2023 Nov 20;19(11):e1011627. doi: 10.1371/journal.pcbi.1011627 (PMC10695395; doi:10.1371/journal.pcbi.1011627)
Supplement: S3 Appendix — (PDF) [file pcbi.1011627.s003.pdf]

Imaging with spatio-temporal modelling to characterize the dynamics of plant-pathogen lesions
Appendix S3

Melen Leclerc[1], Stéphane Jumel[1], Frédéric M. Hamelin[1], Rémi Treilhaud[1], Nicolas Parisey[1], and Youcef Mammeri[2]

[1]IGEPP, INRAE, Institut Agro, University of Rennes, Rennes, France
[2]ICJ, CNRS, Jean Monnet University, Saint-Etienne, France

- S3.1 Probability images, fitted models and images of residuals for all 32 inoculated stipules (p 2 - 33).
- S3.2 Boxplots showing the distributions of residuals for all dates and inoculated stipules (p 34 - 35).
- S3.3 Comparison of predicted infection probability against probability given by classifiers (pixel versus pixel) for all inoculated stipules (p 36 - 37).

S3 Assessment of the discrepancy between model and data

S3.1 Spatial comparison of model prediction against data

For each monitored inoculated stipules the discrepancy between the probability images (top) and the fitted reaction-diffusion model (middle) is assessed by visualizing the raw residuals $[u_{reg}(\mathbf{x}, t_i) - u(\mathbf{x}, t_i, \hat{\theta})]$ (bottom) at day 4, 5, 6 and 7 after inoculation (from left to right).

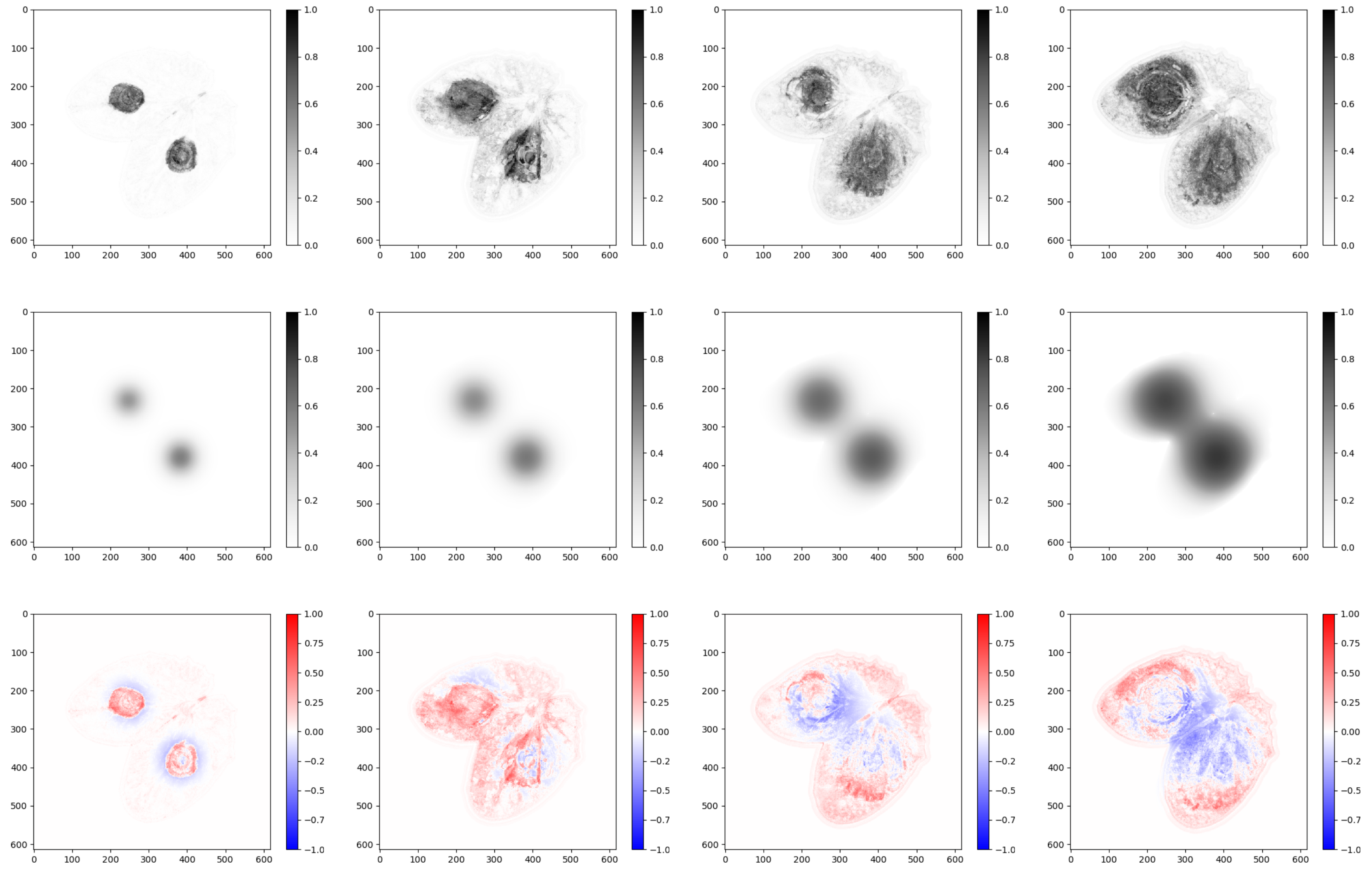

Figure A: Solara N°1

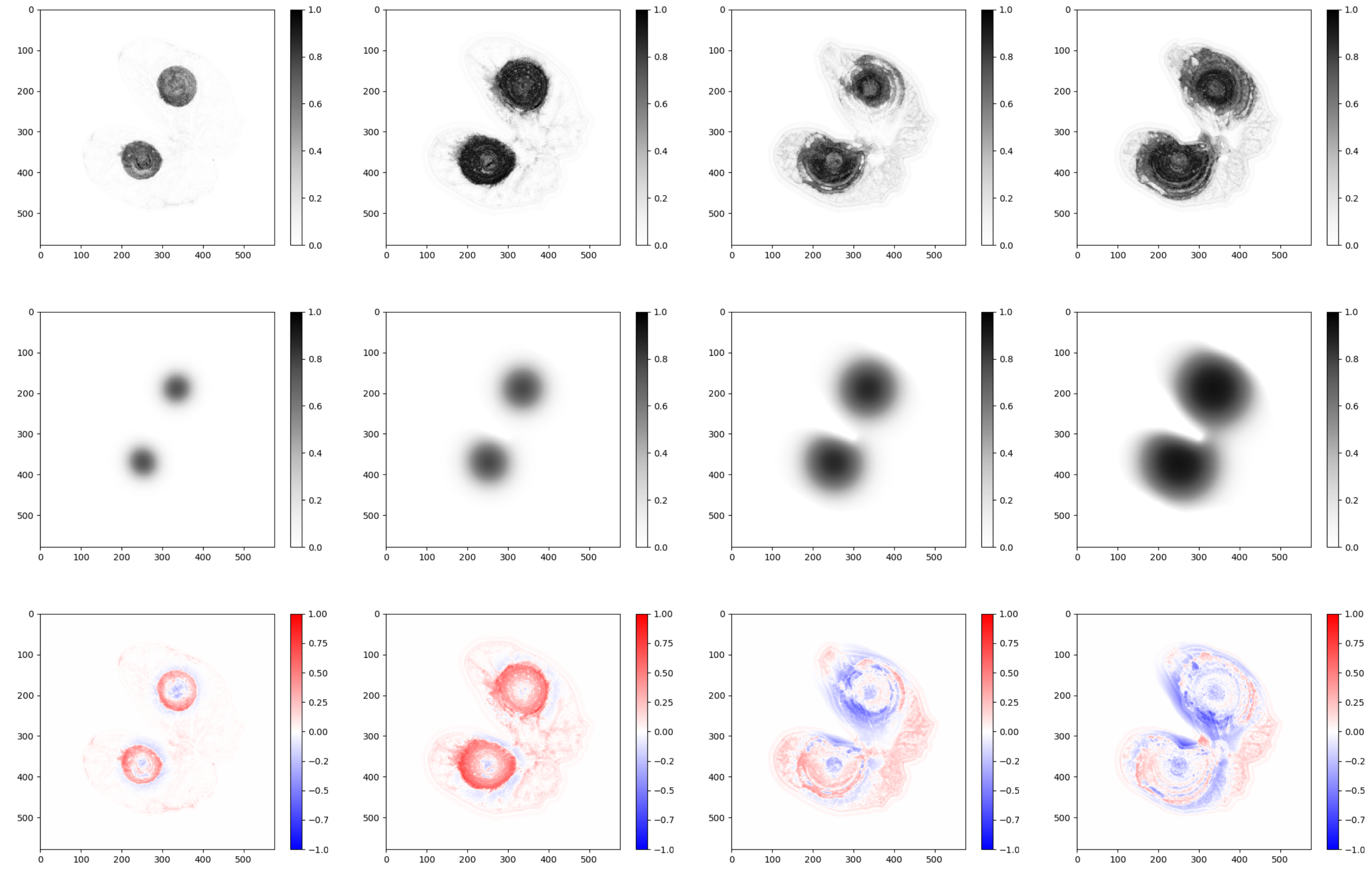

Figure B: Solara N°2

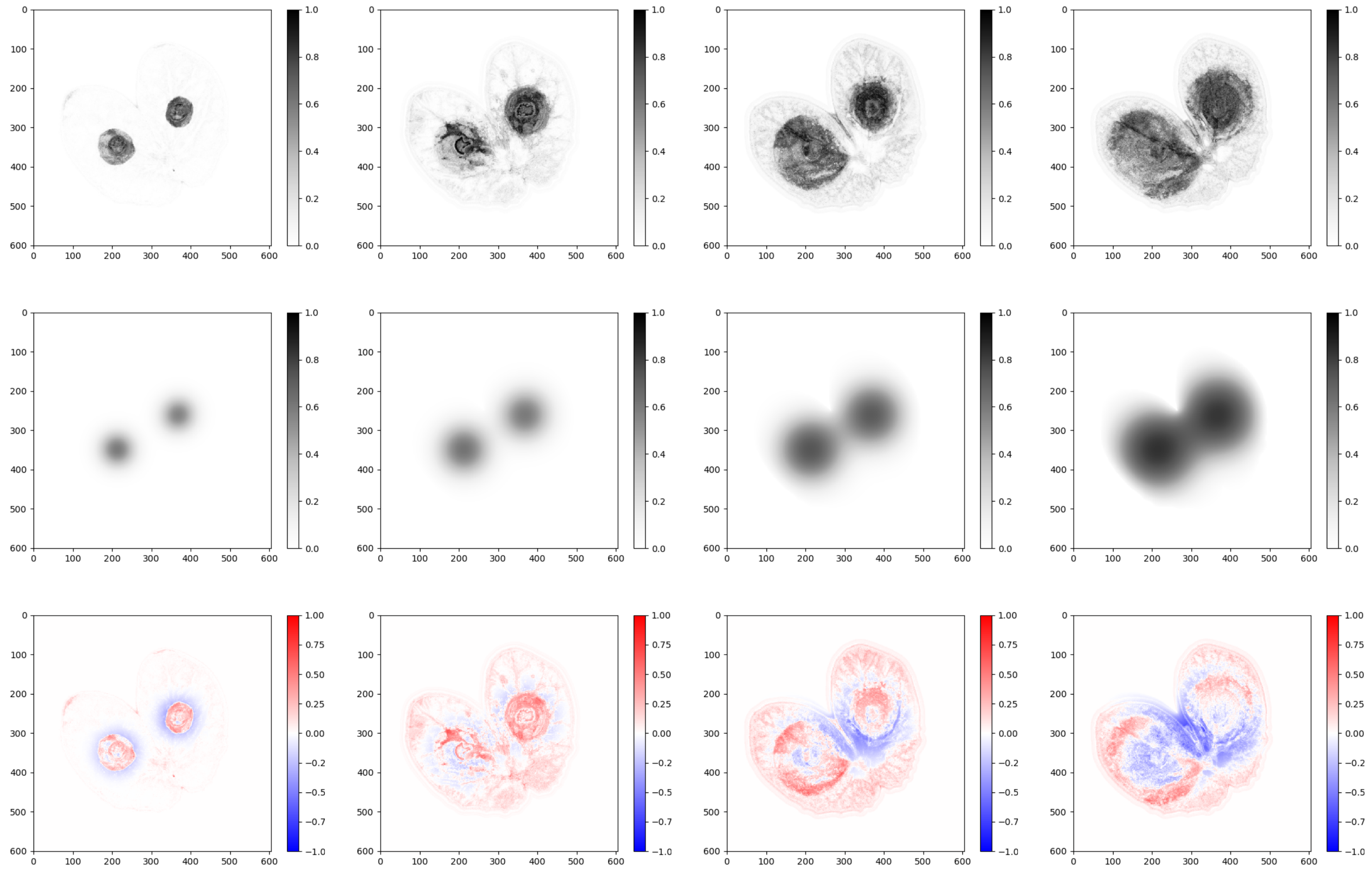

Figure C: Solara N°3

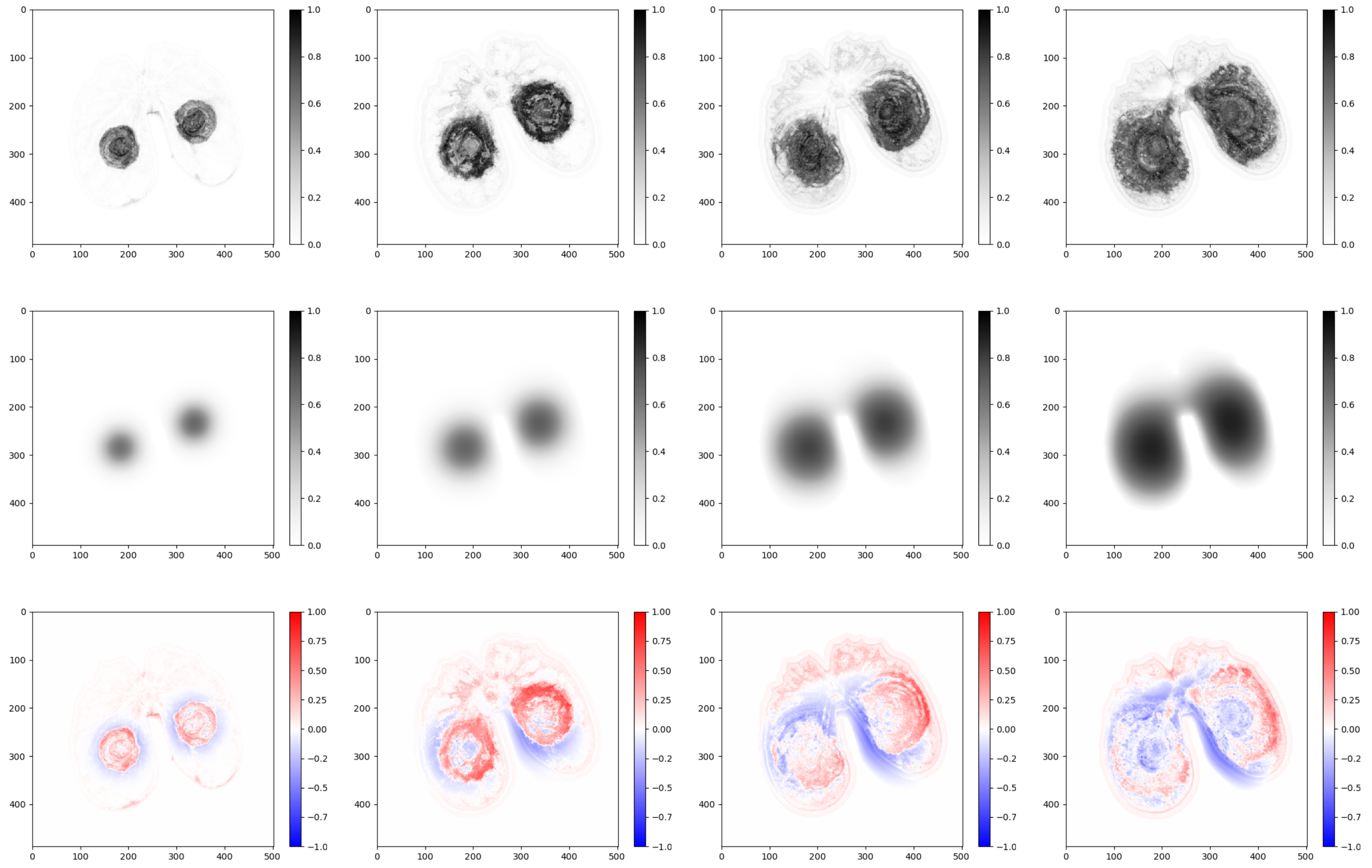

Figure D: Solara N°4

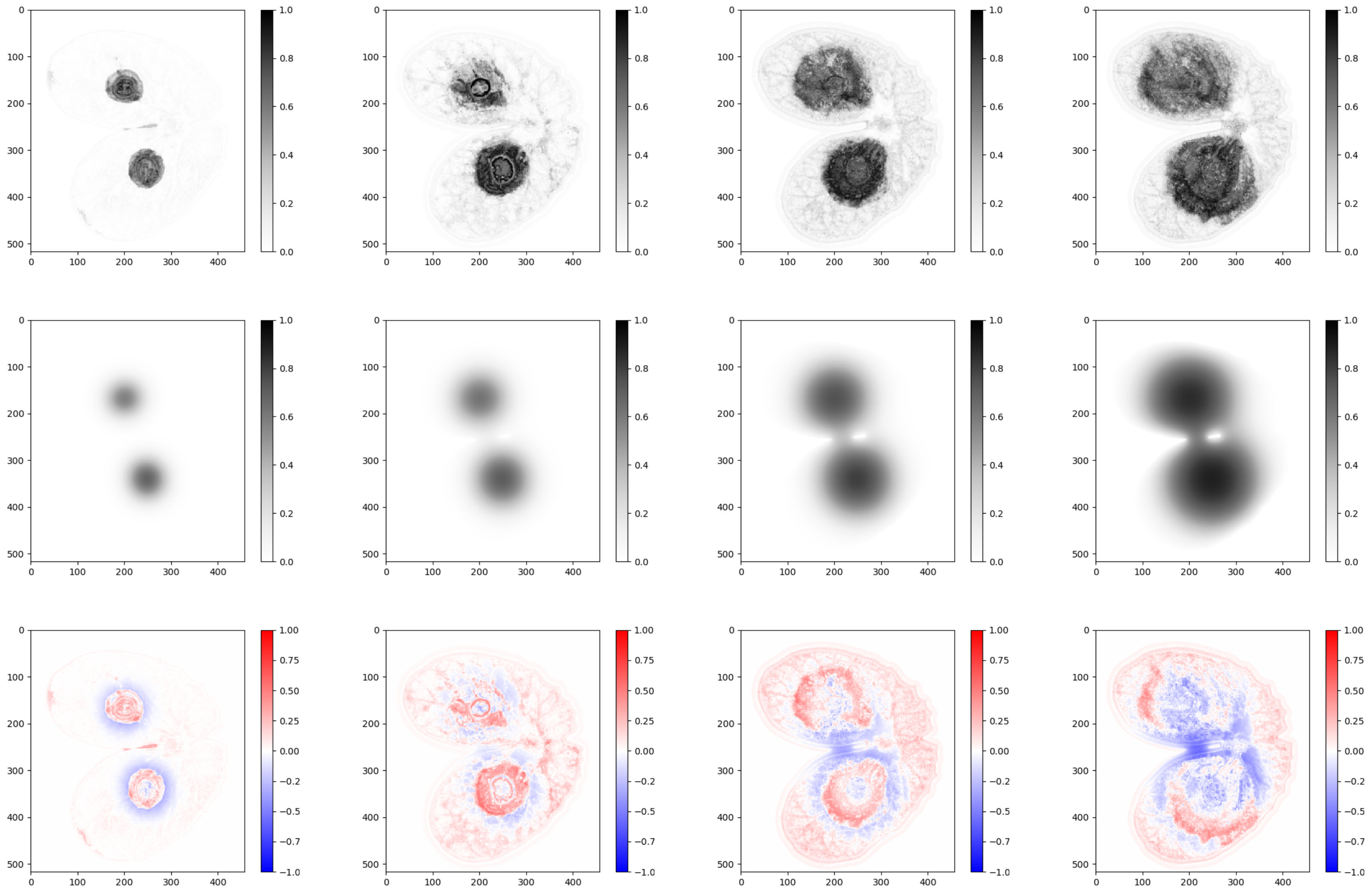

Figure E: Solara N°5

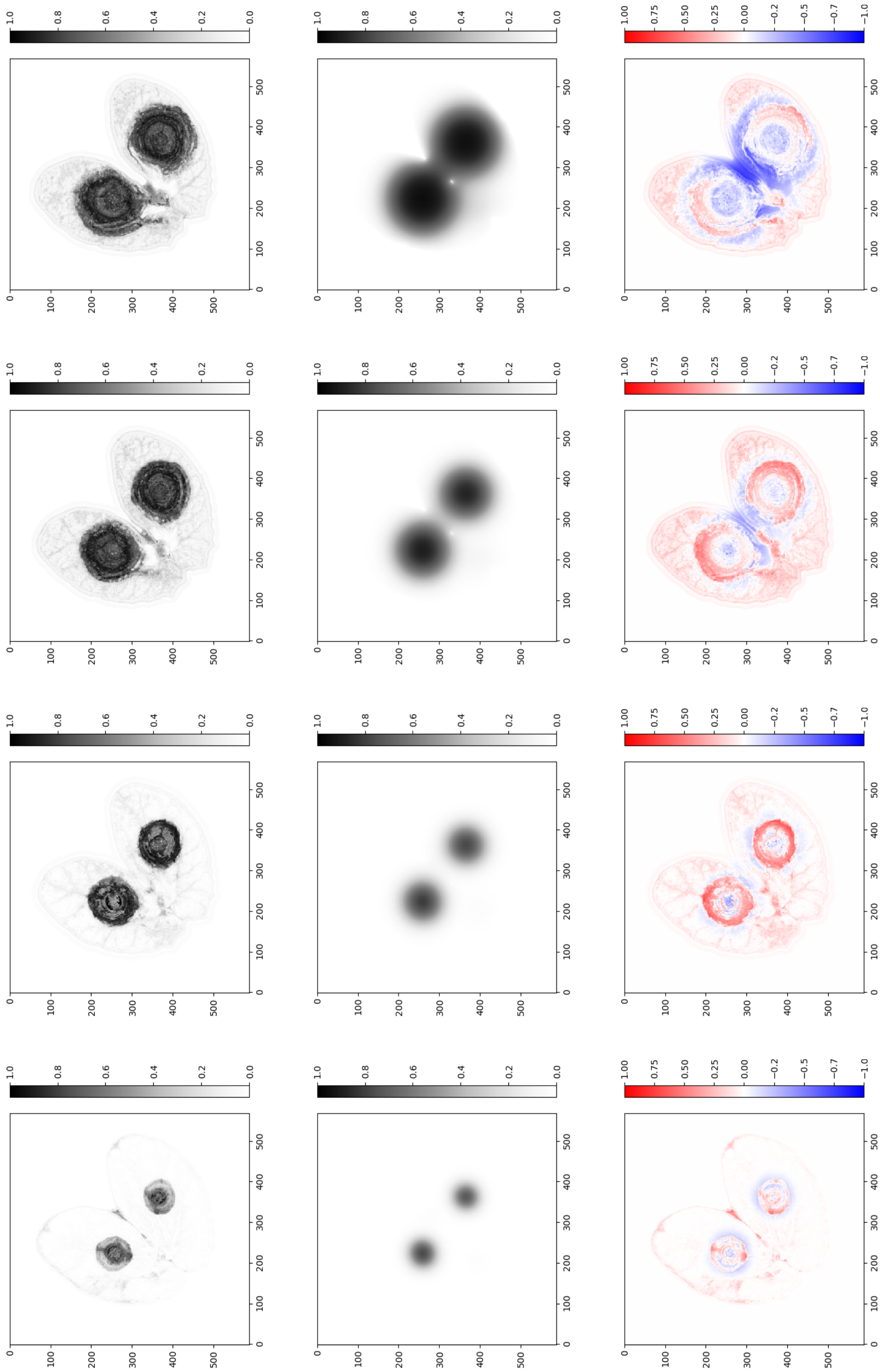

Figure F: Solara N°6

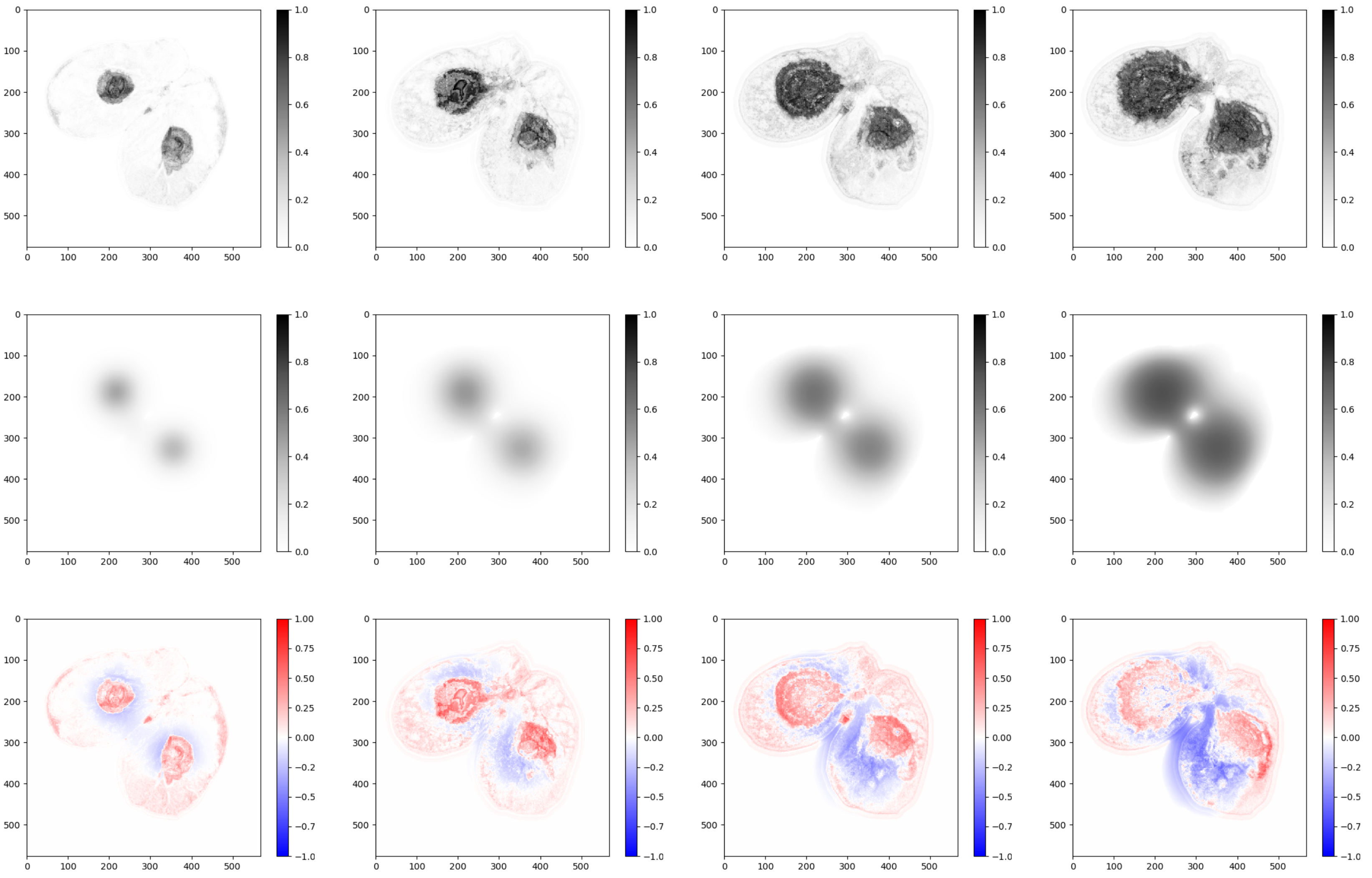

Figure G: Solara N°7

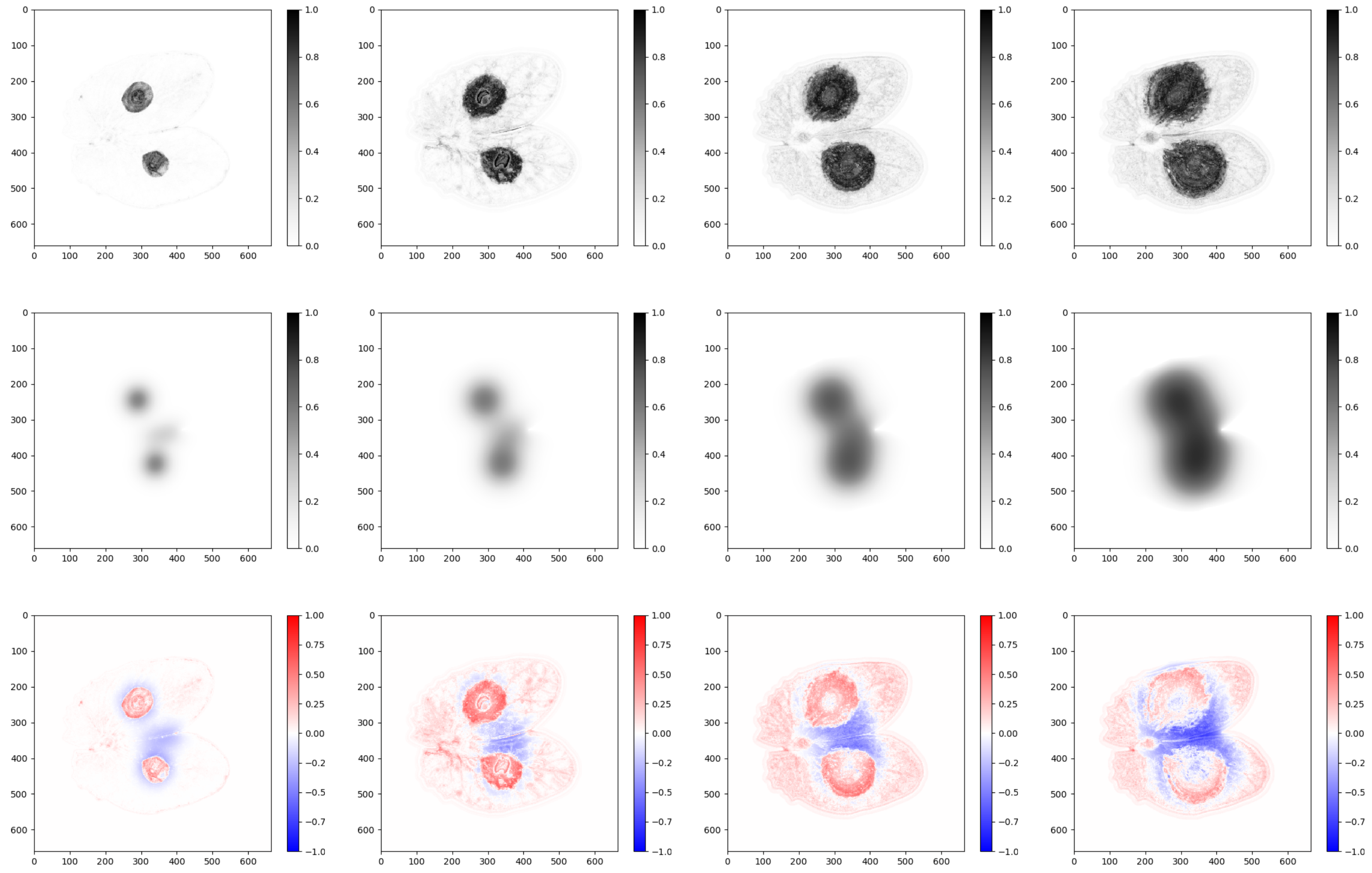

Figure H: Solara N°8

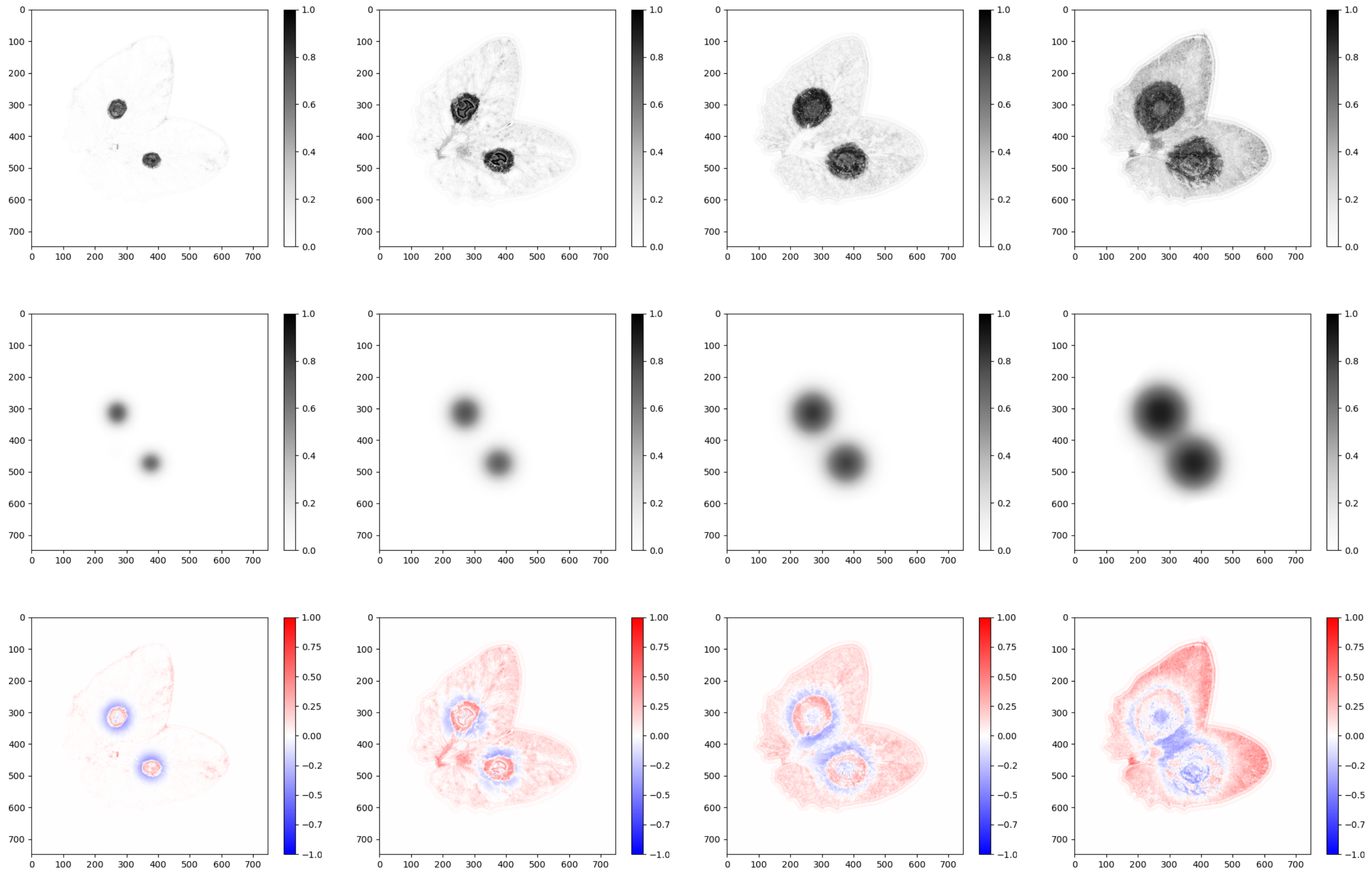

Figure I: Solara N°9

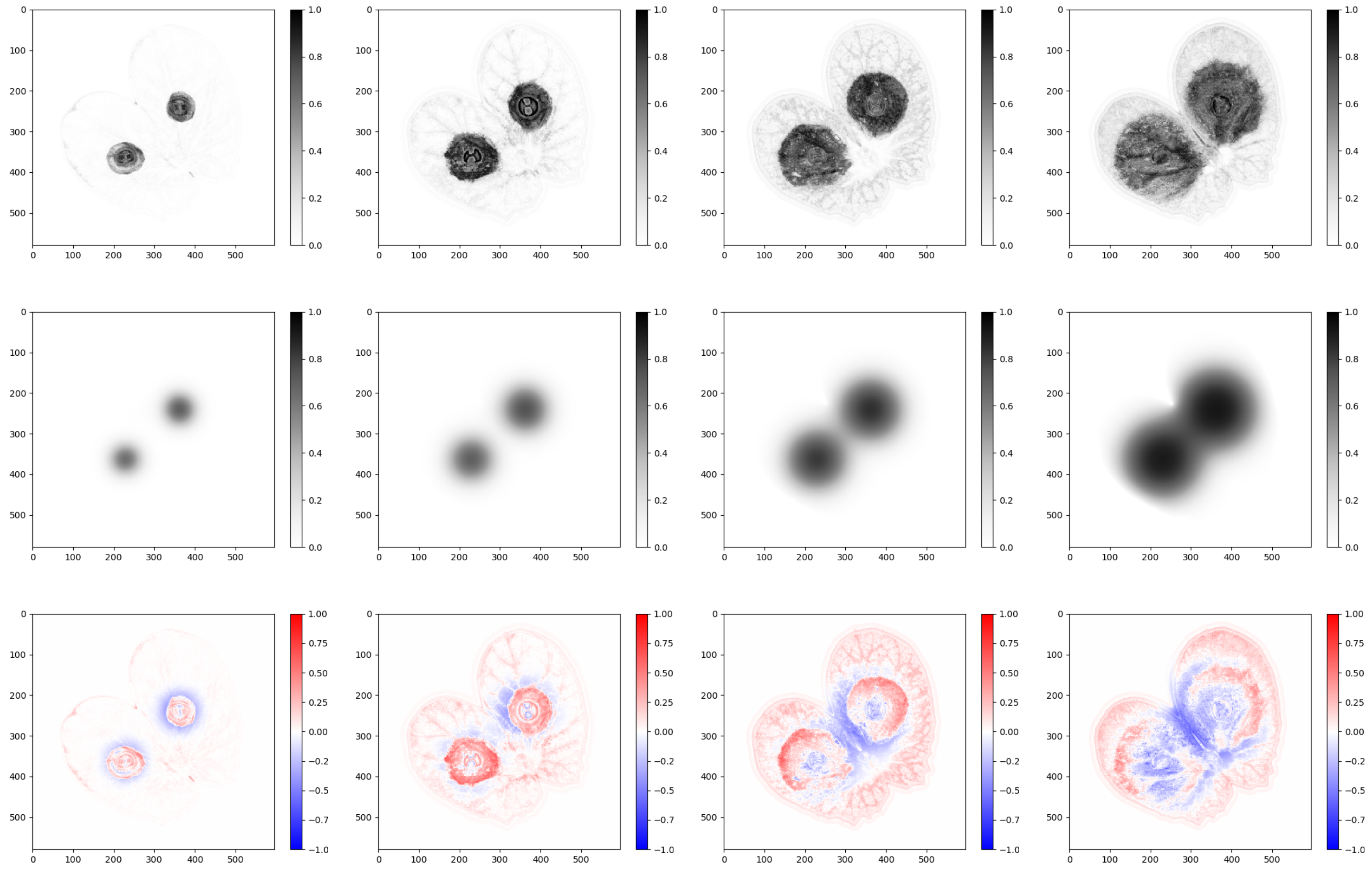

Figure J: Solara N°10

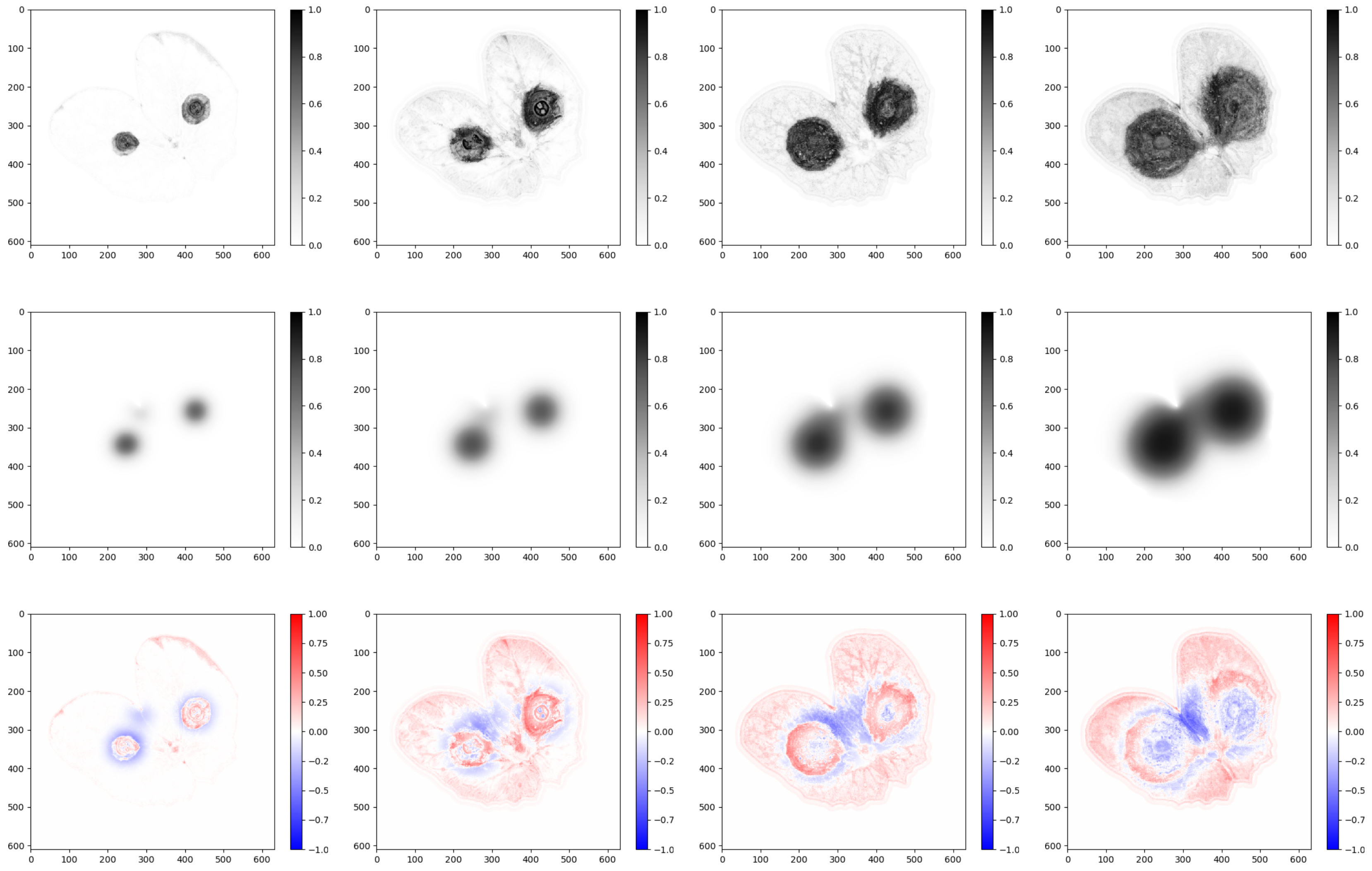

Figure K: Solara N°11

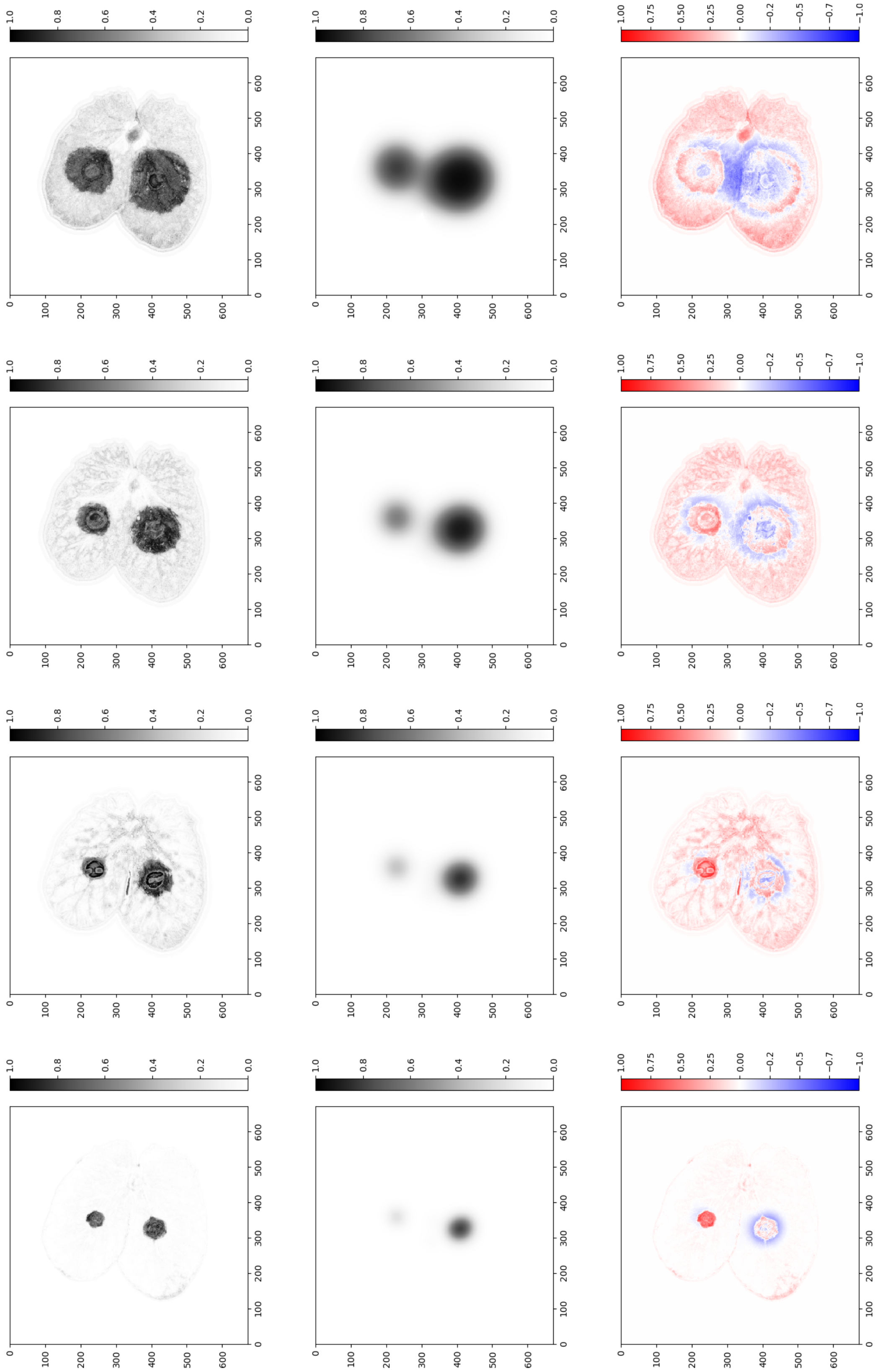

Figure L: Solara N°12

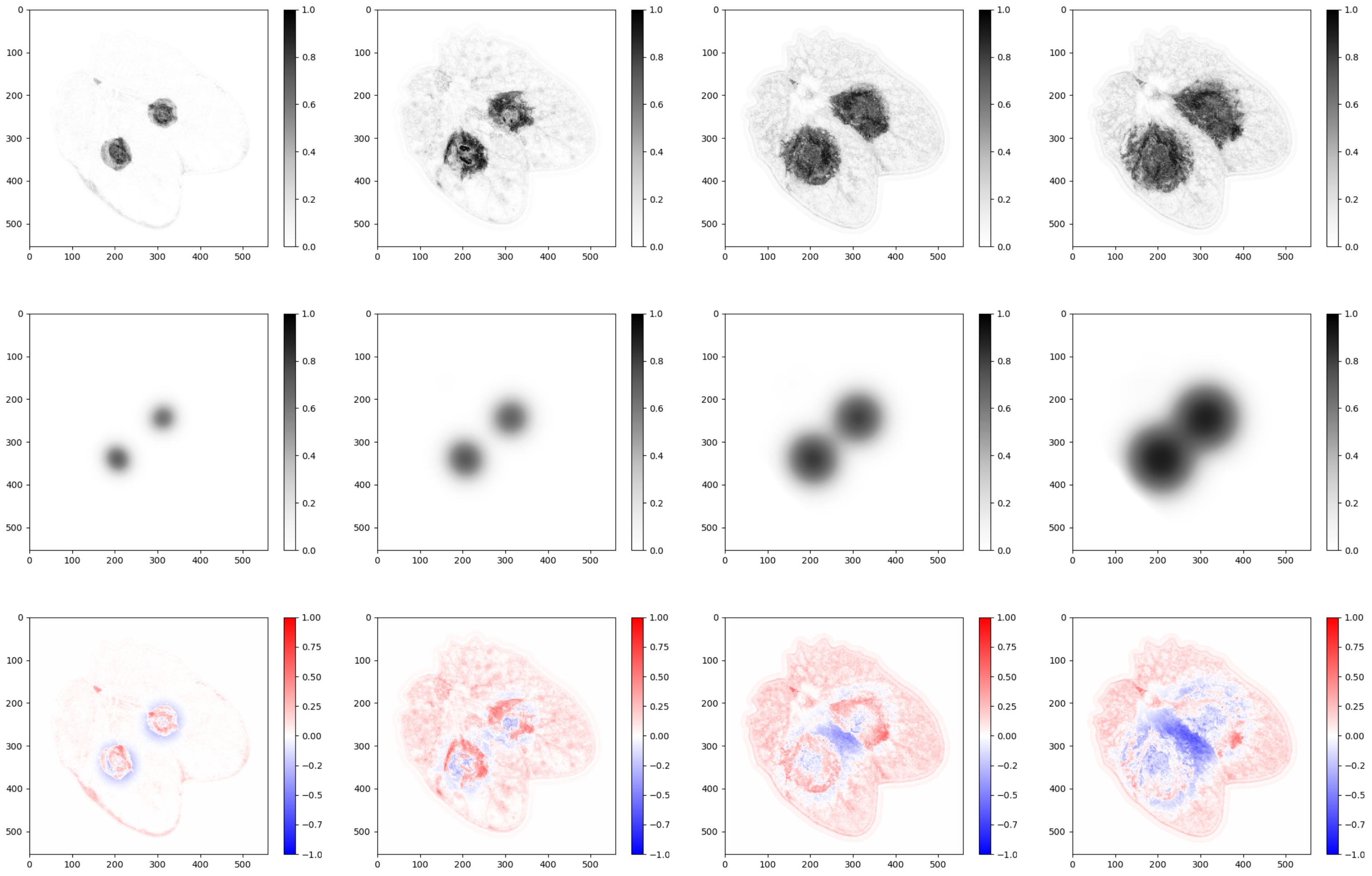

Figure M: Solara N°13

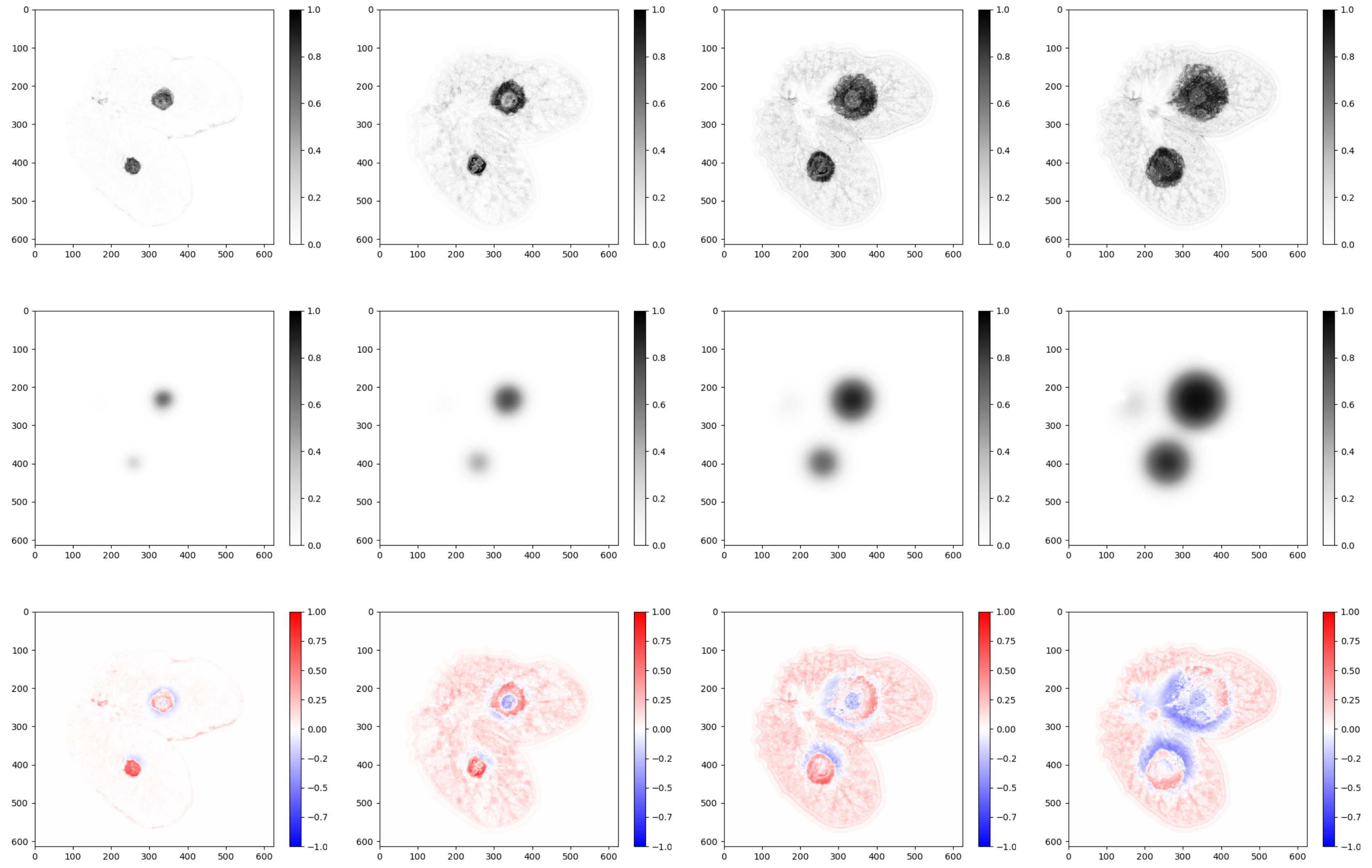

Figure N: Solara N°14

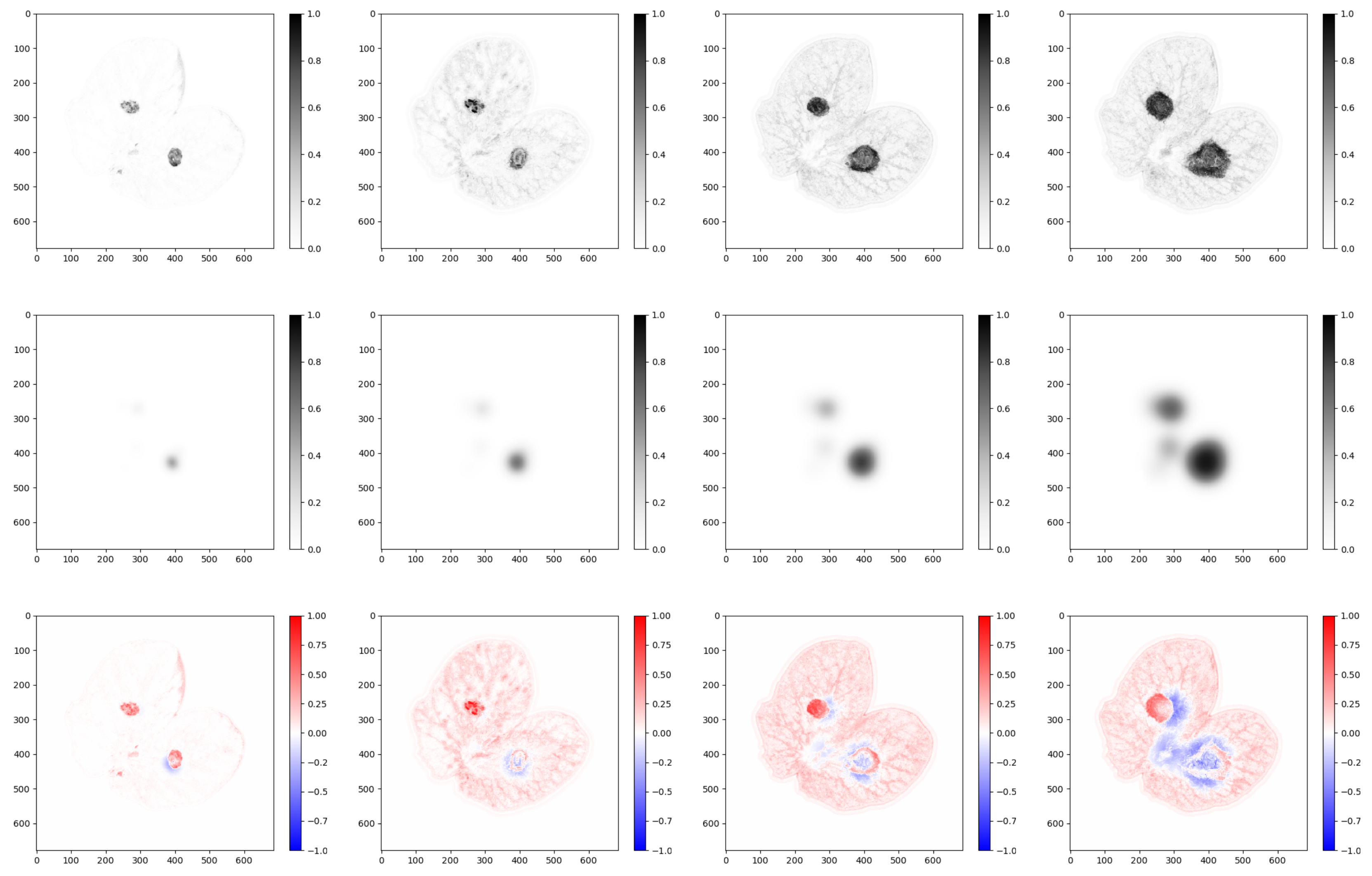

Figure O: Solara N°15

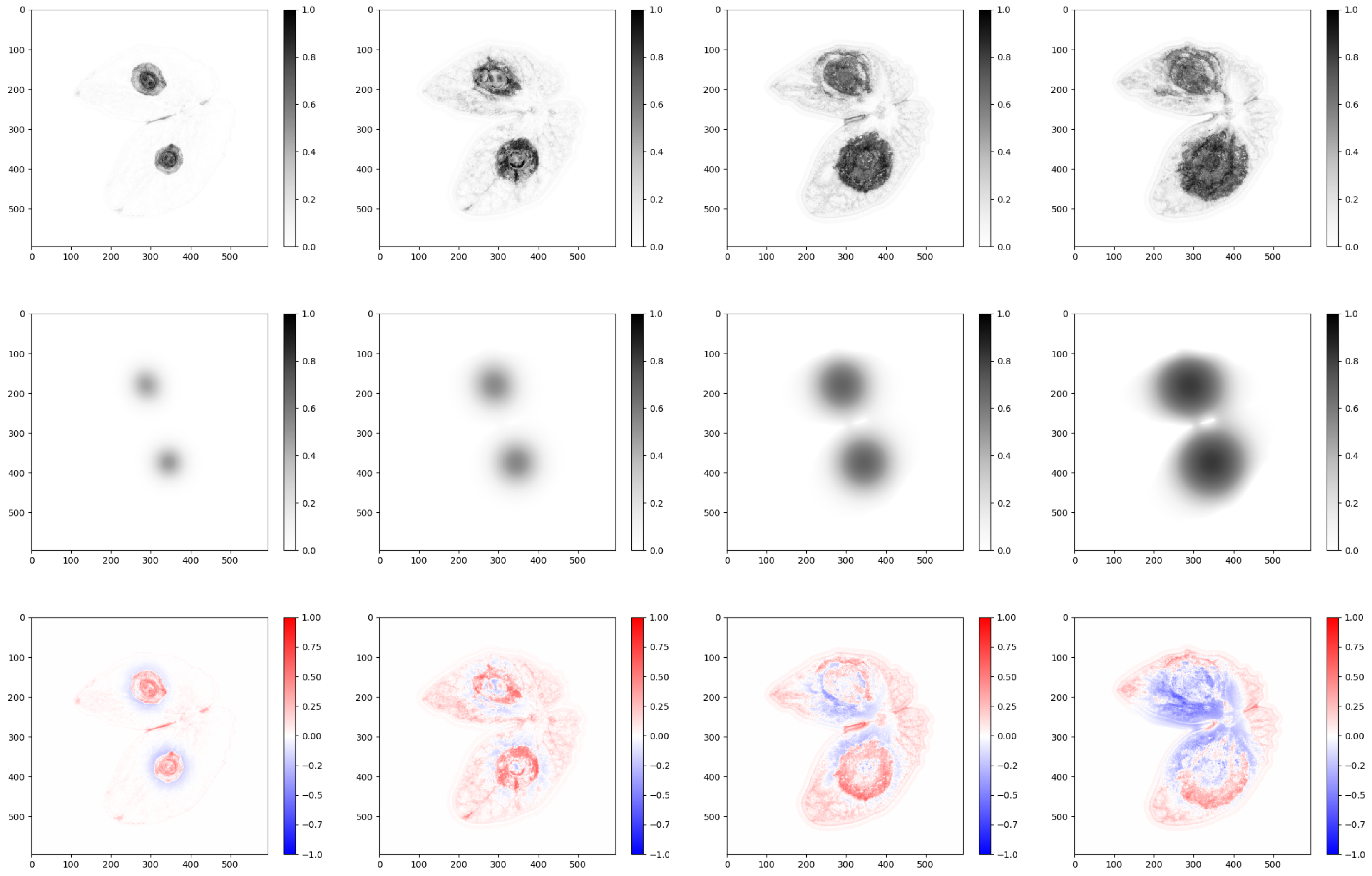

Figure P: Solara N°16

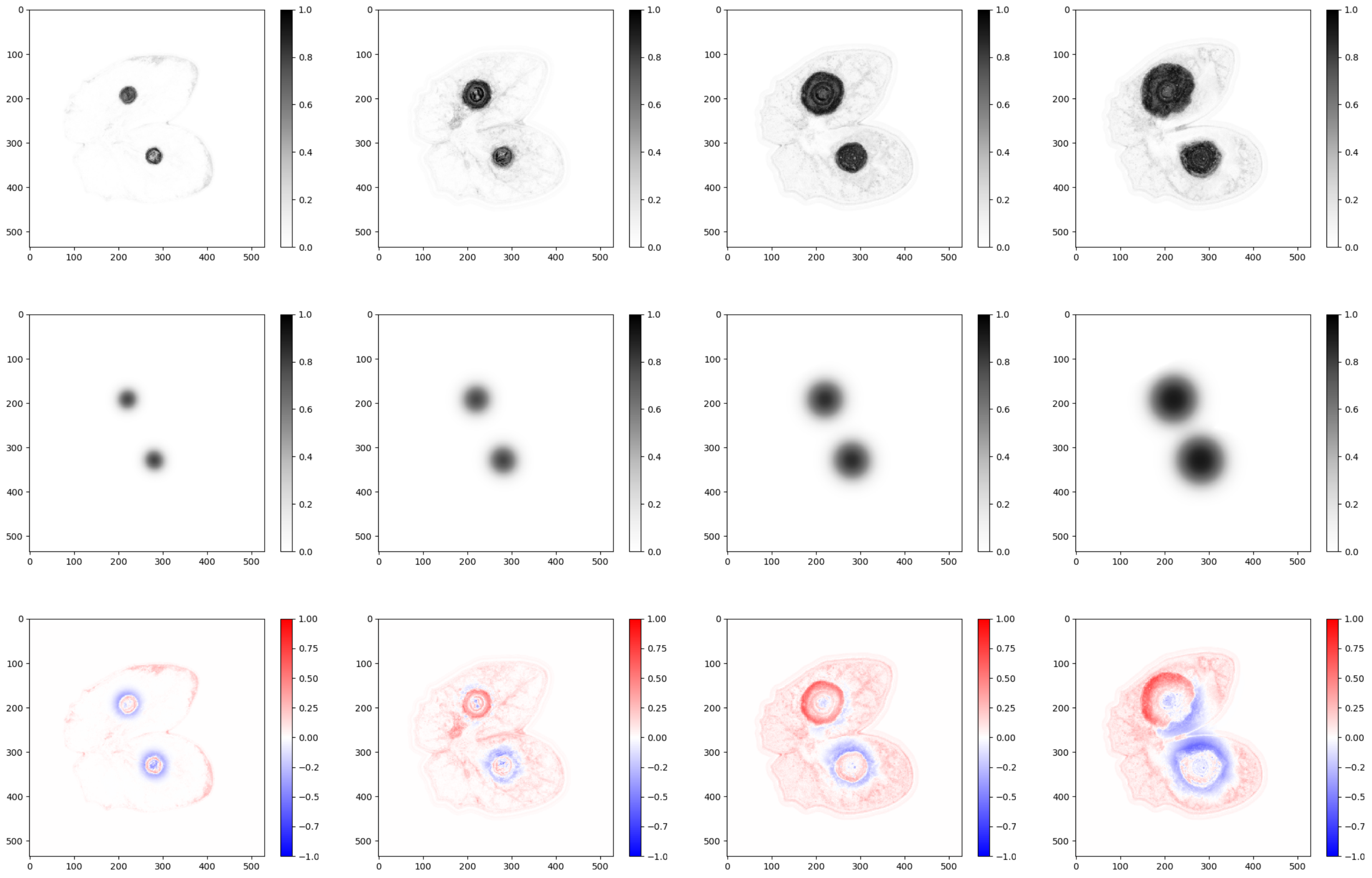

Figure Q: James N°17

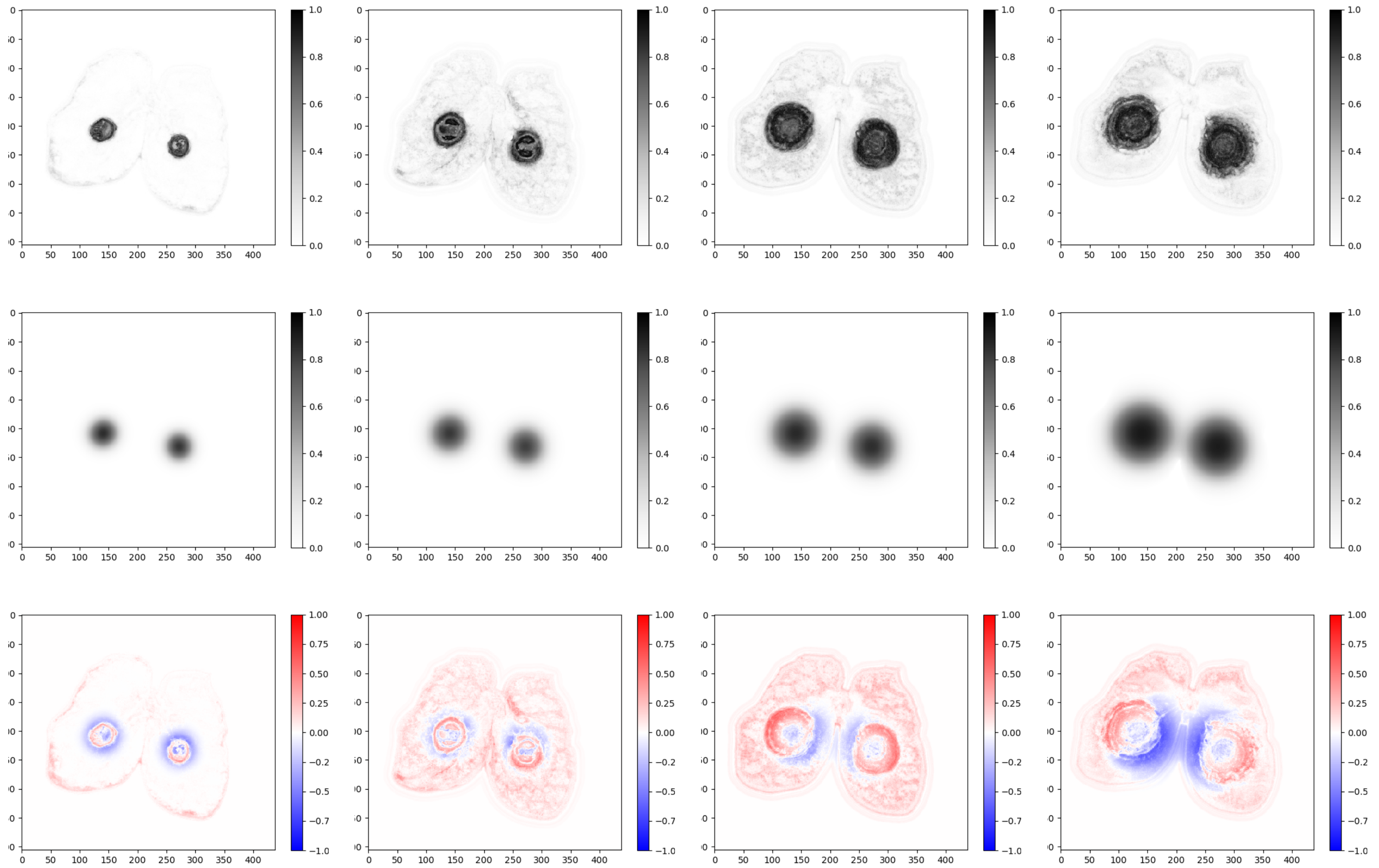

Figure R: James N°18

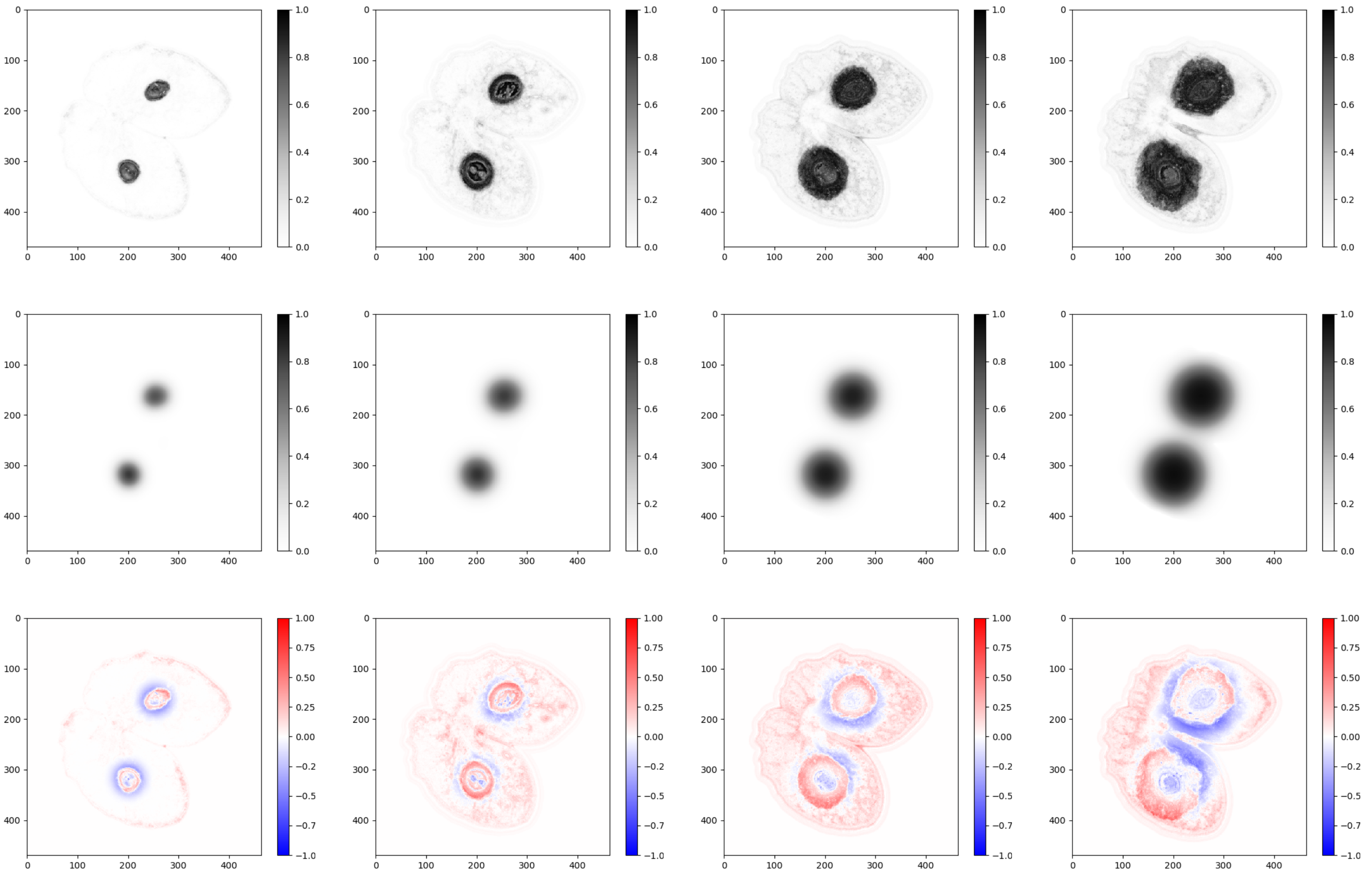

Figure S: James N°19

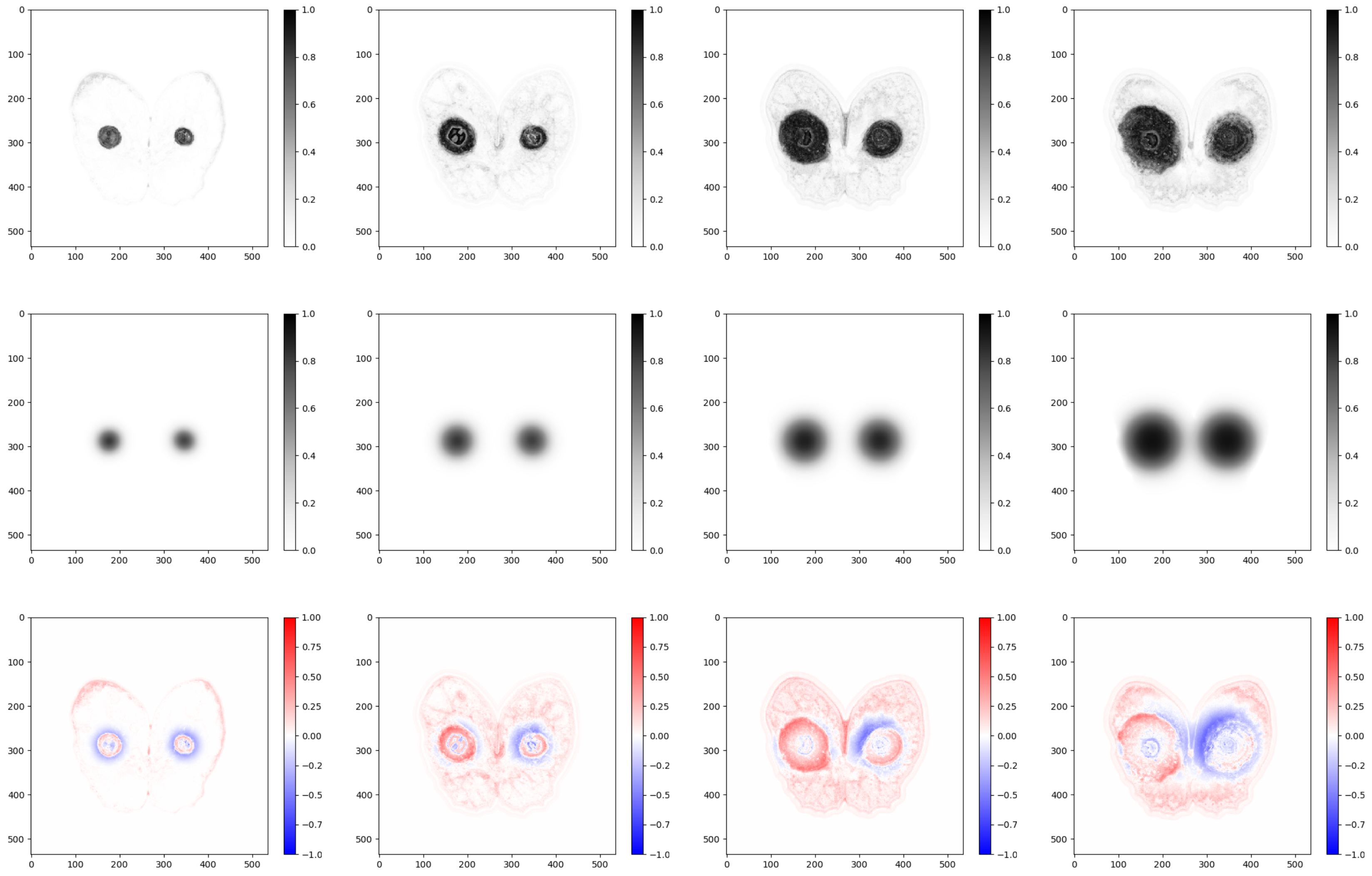

Figure T: James N°20

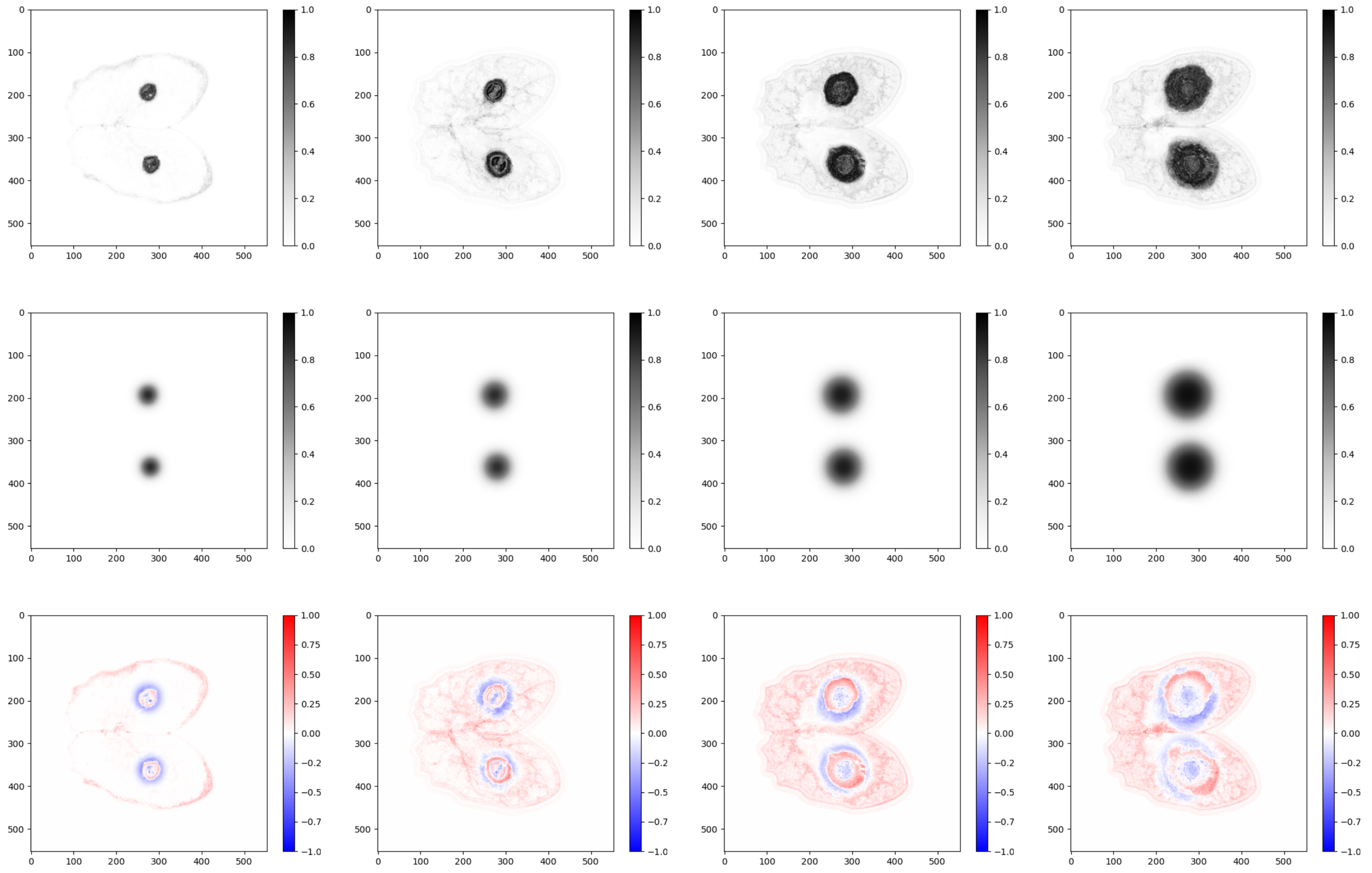

Figure U: James N°21

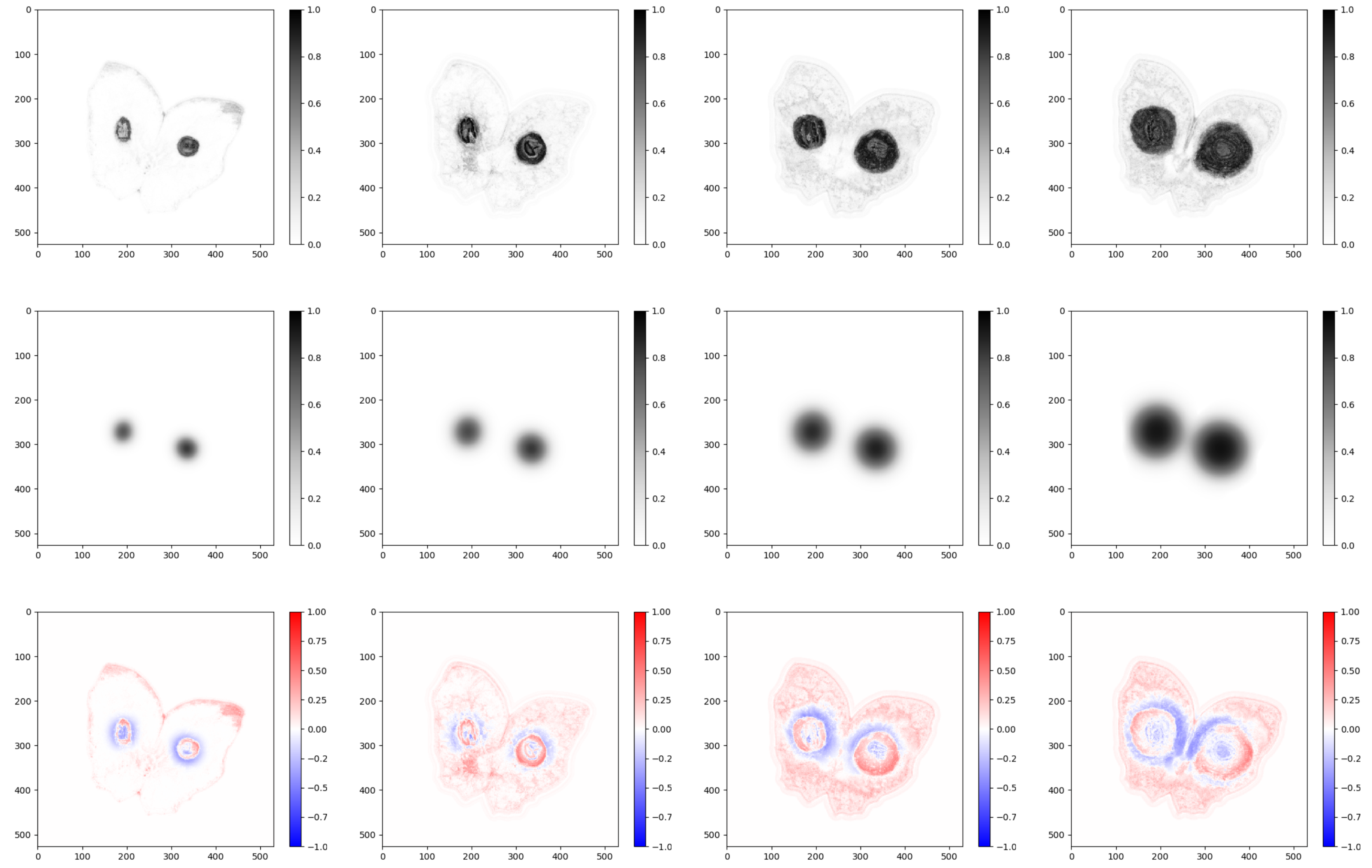

Figure V: James N°22

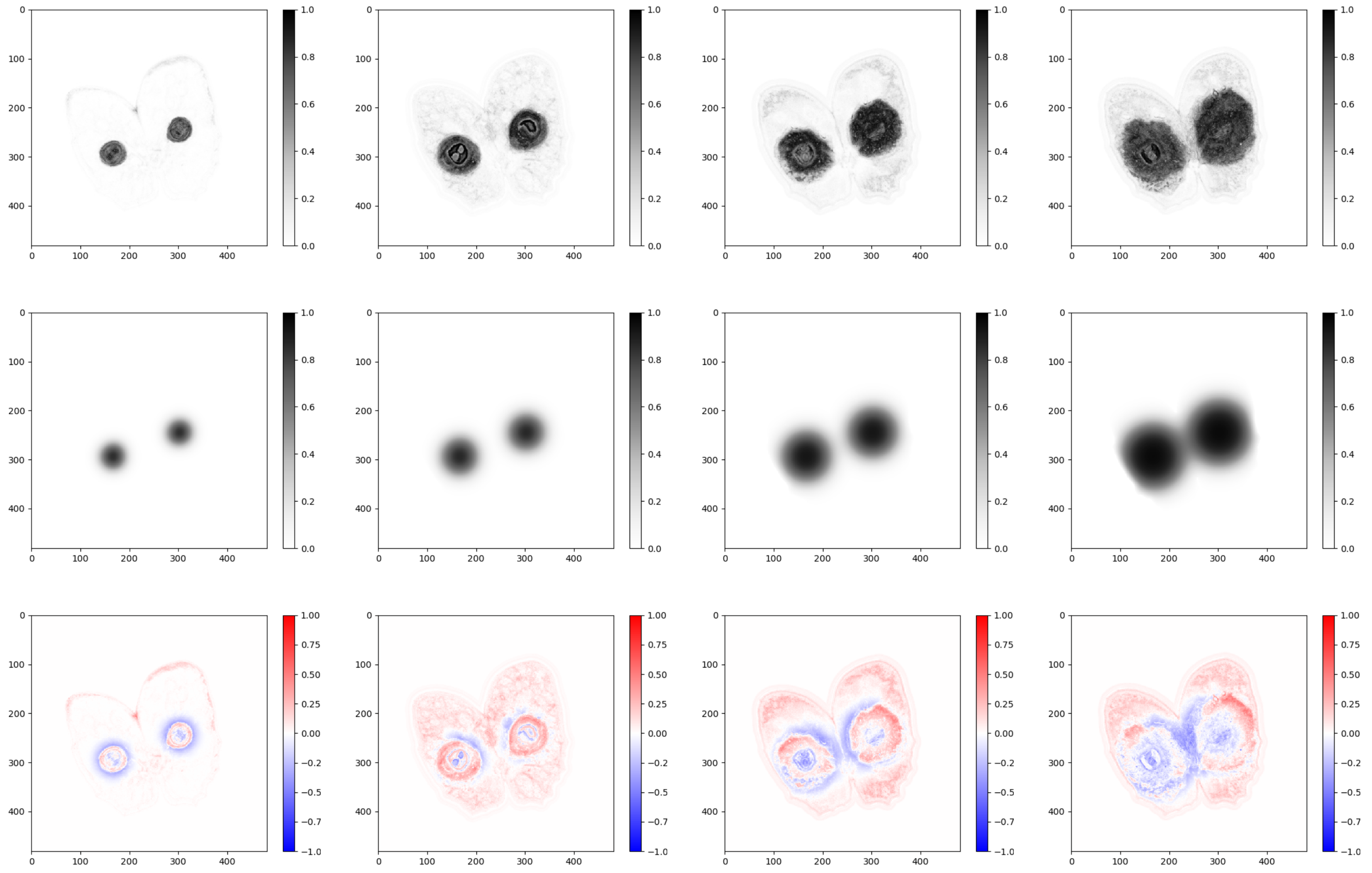

Figure W: James N°23

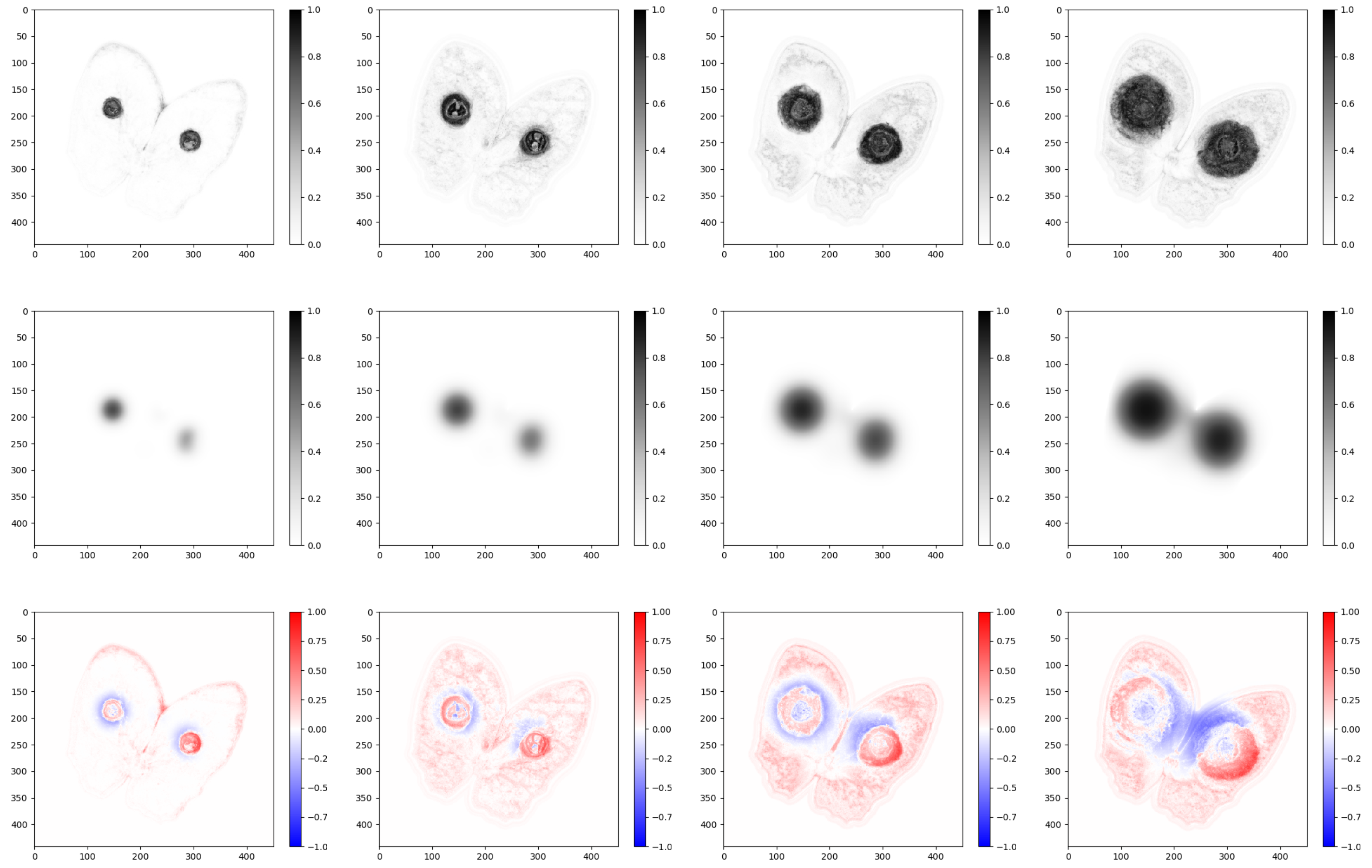

Figure X: James N°24

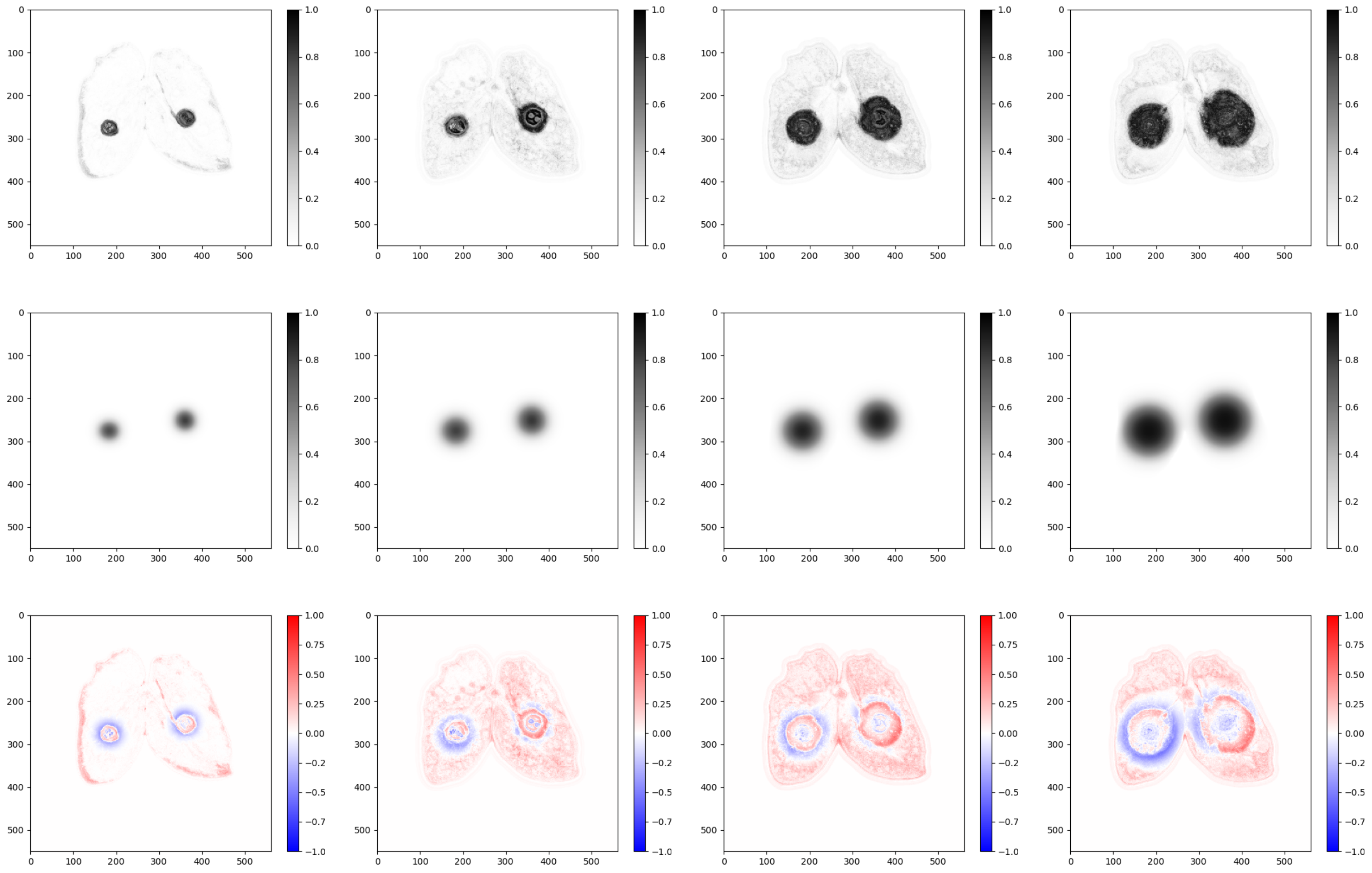

Figure Y: James N°25

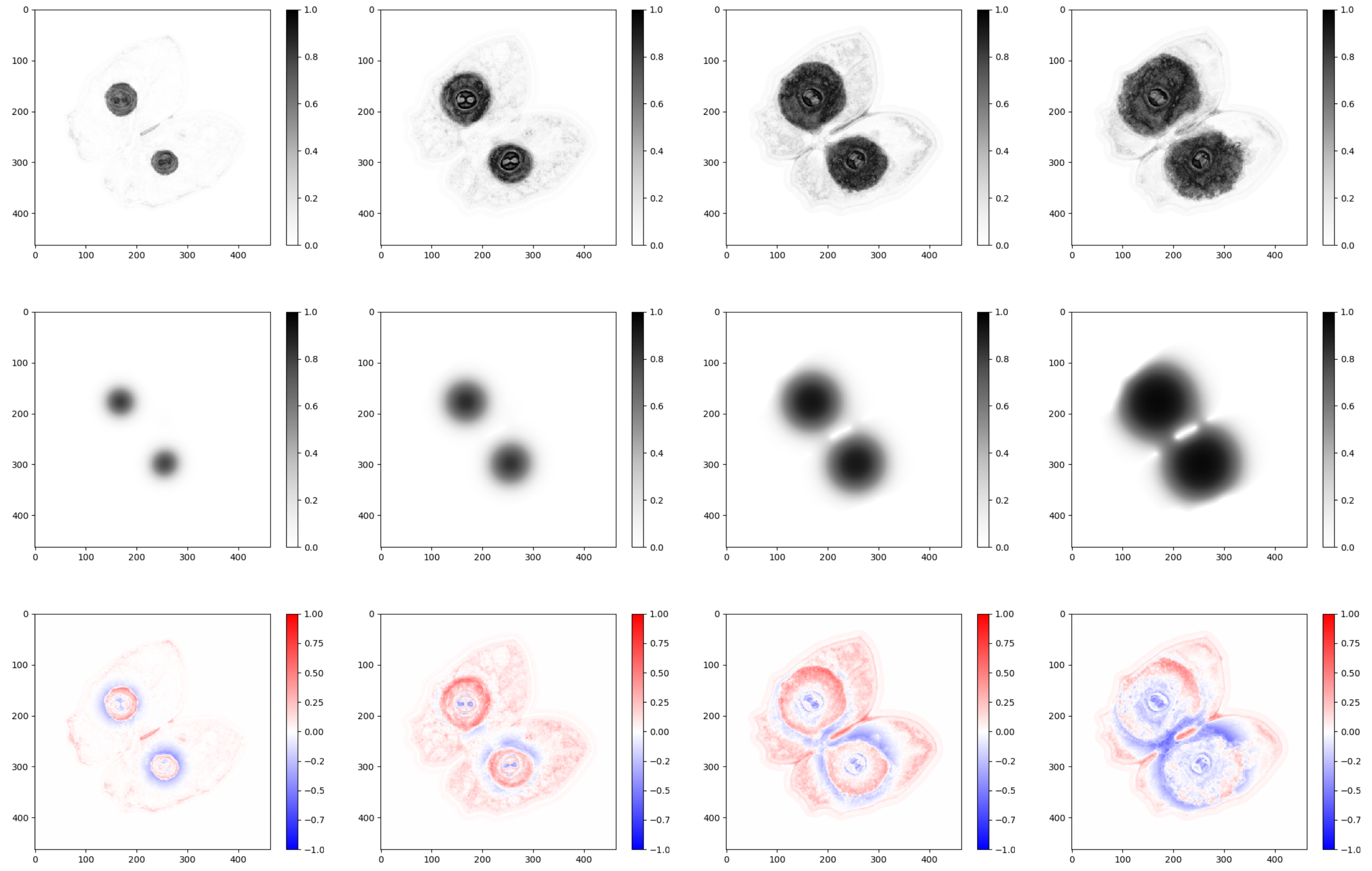

Figure Z: James N°26

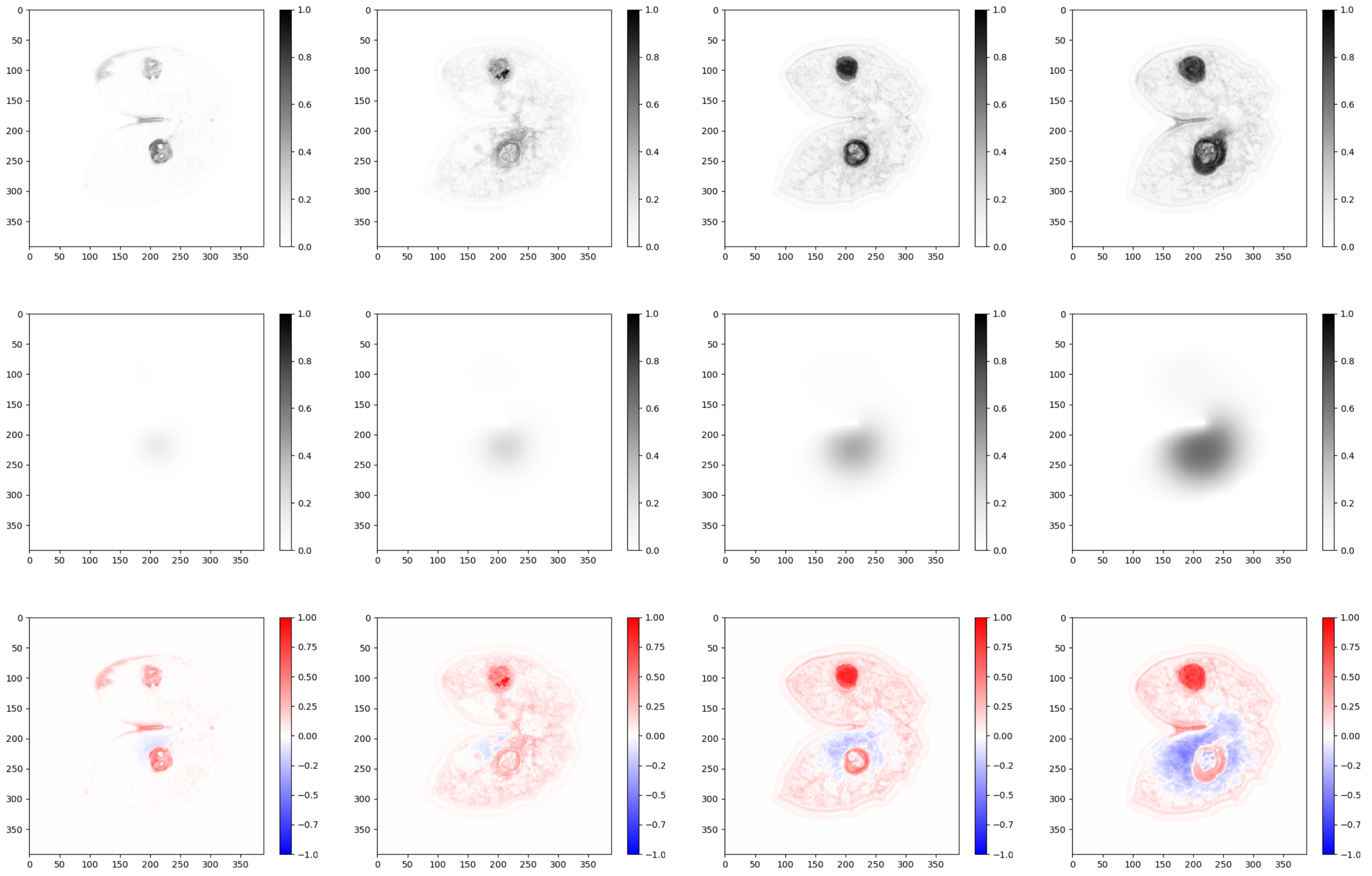

Figure AA: James N°27

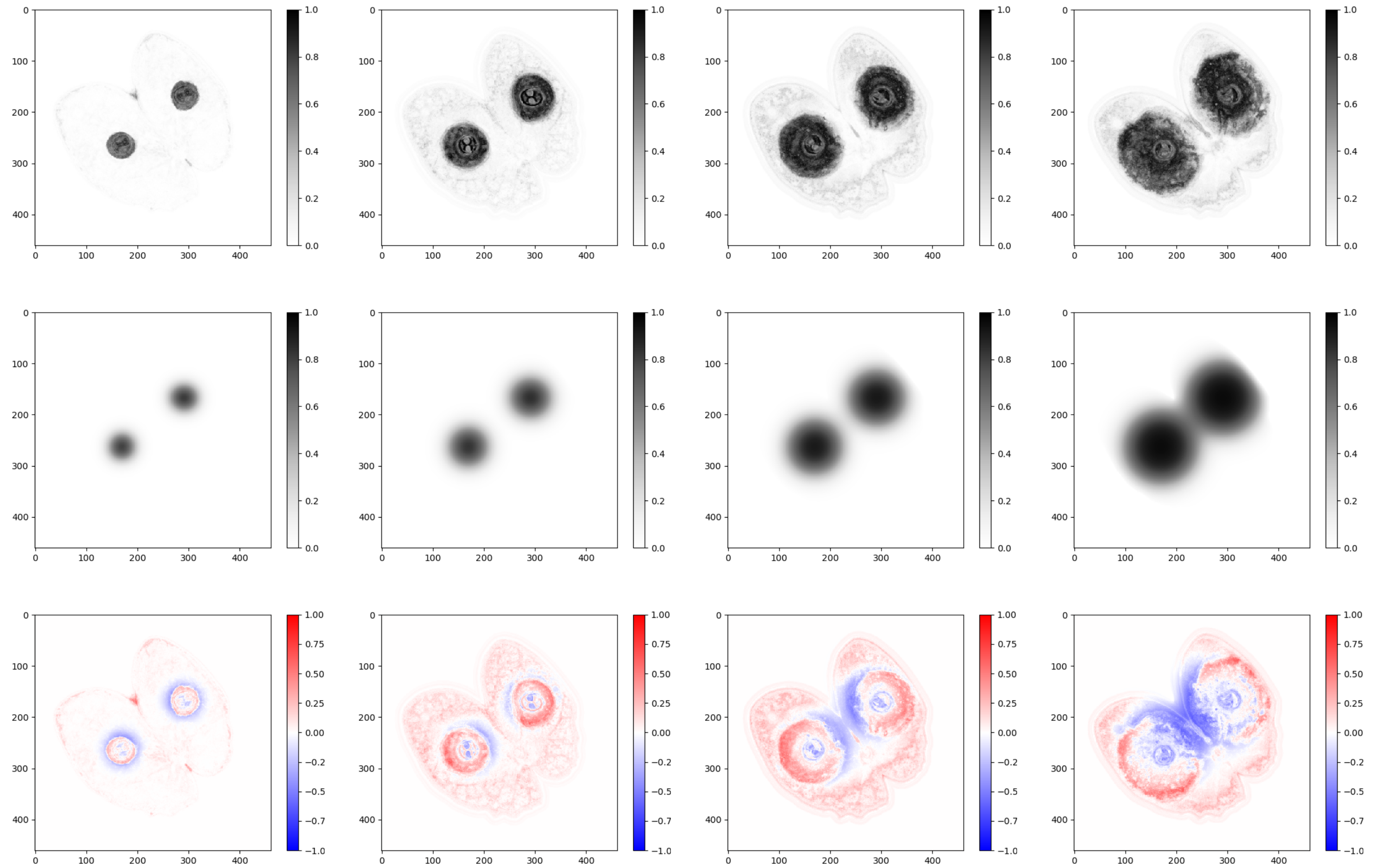

Figure AB: James N°28

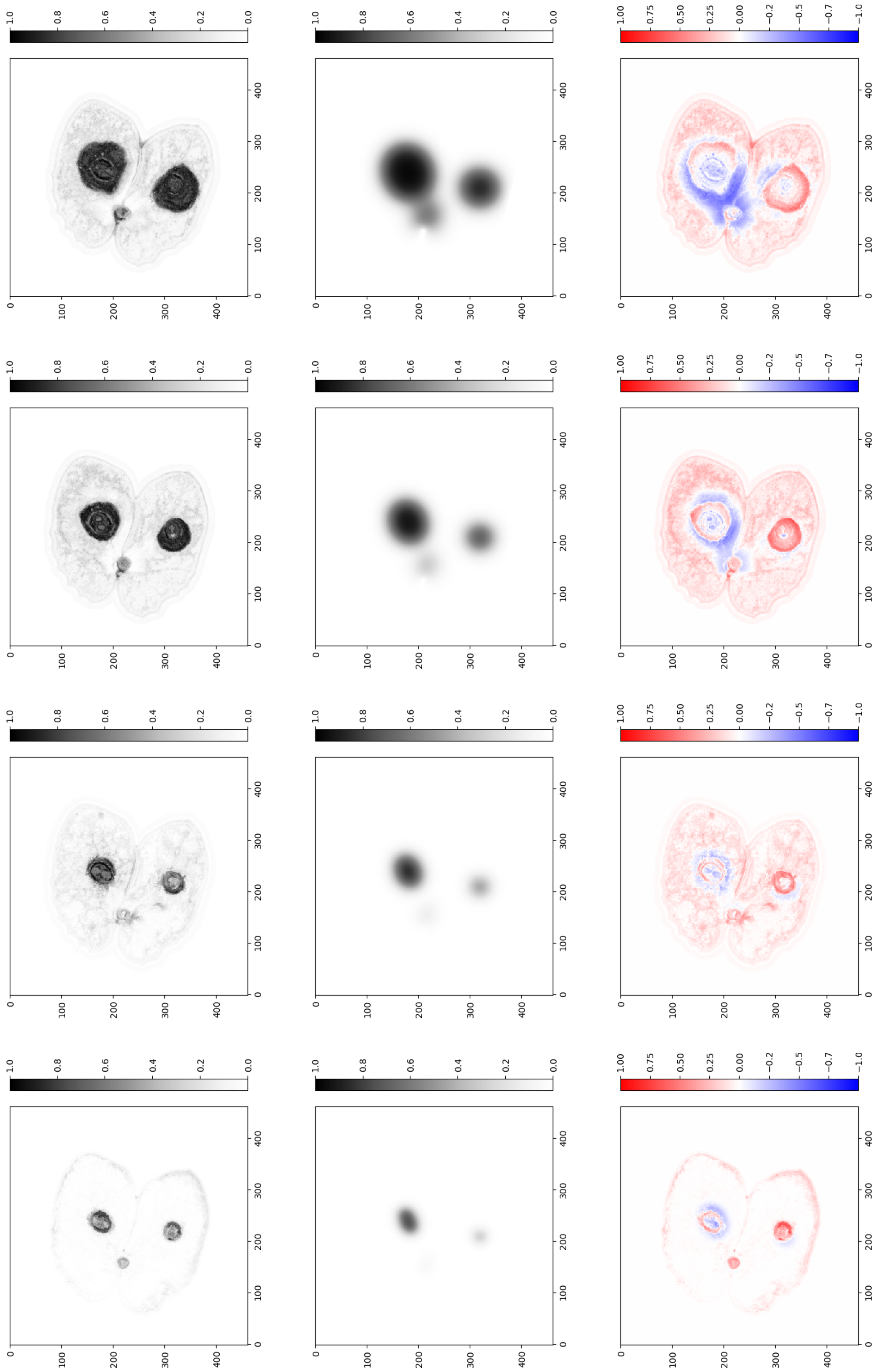

Figure AC: James N°29

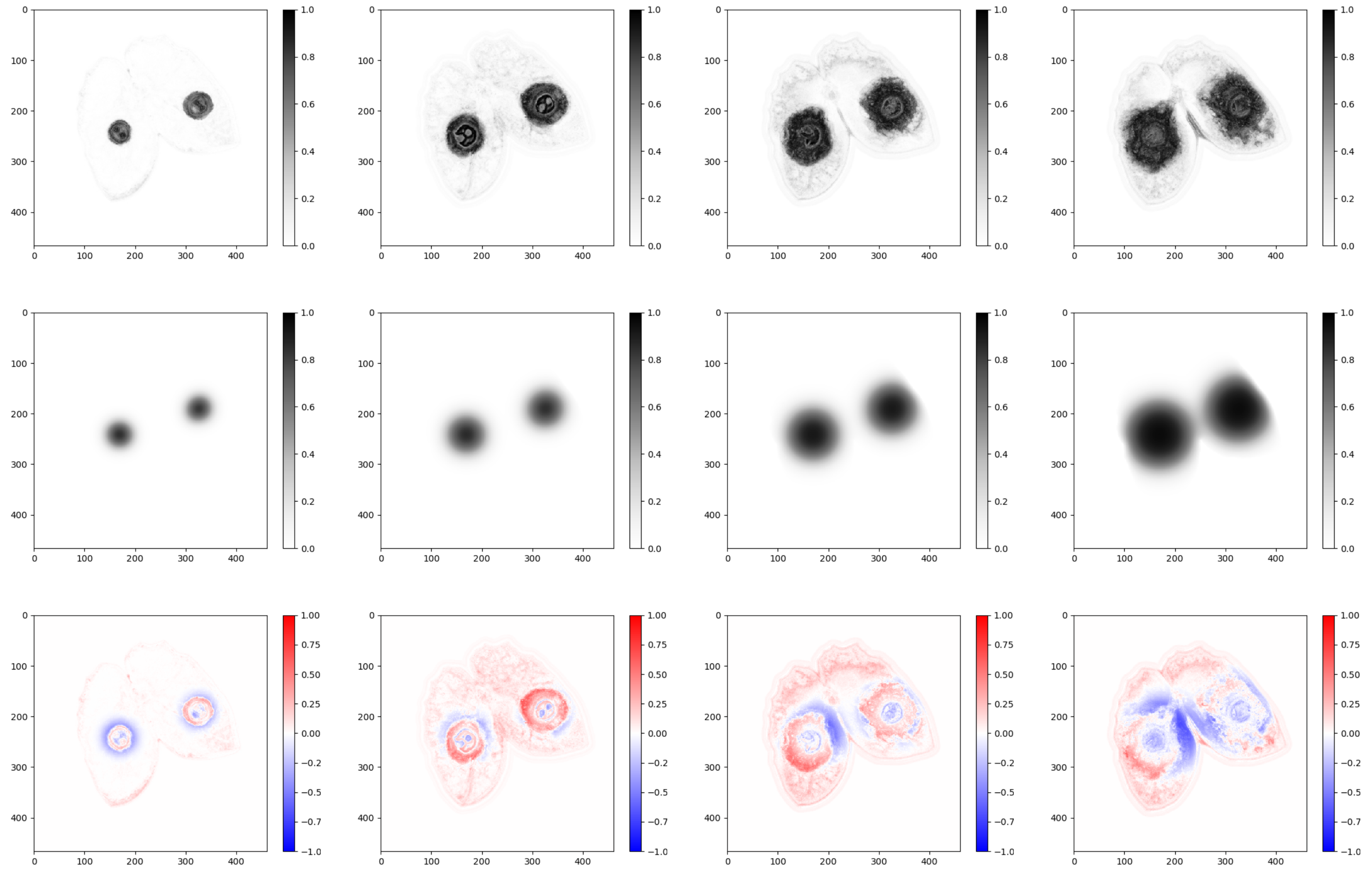

Figure AD: James N°30

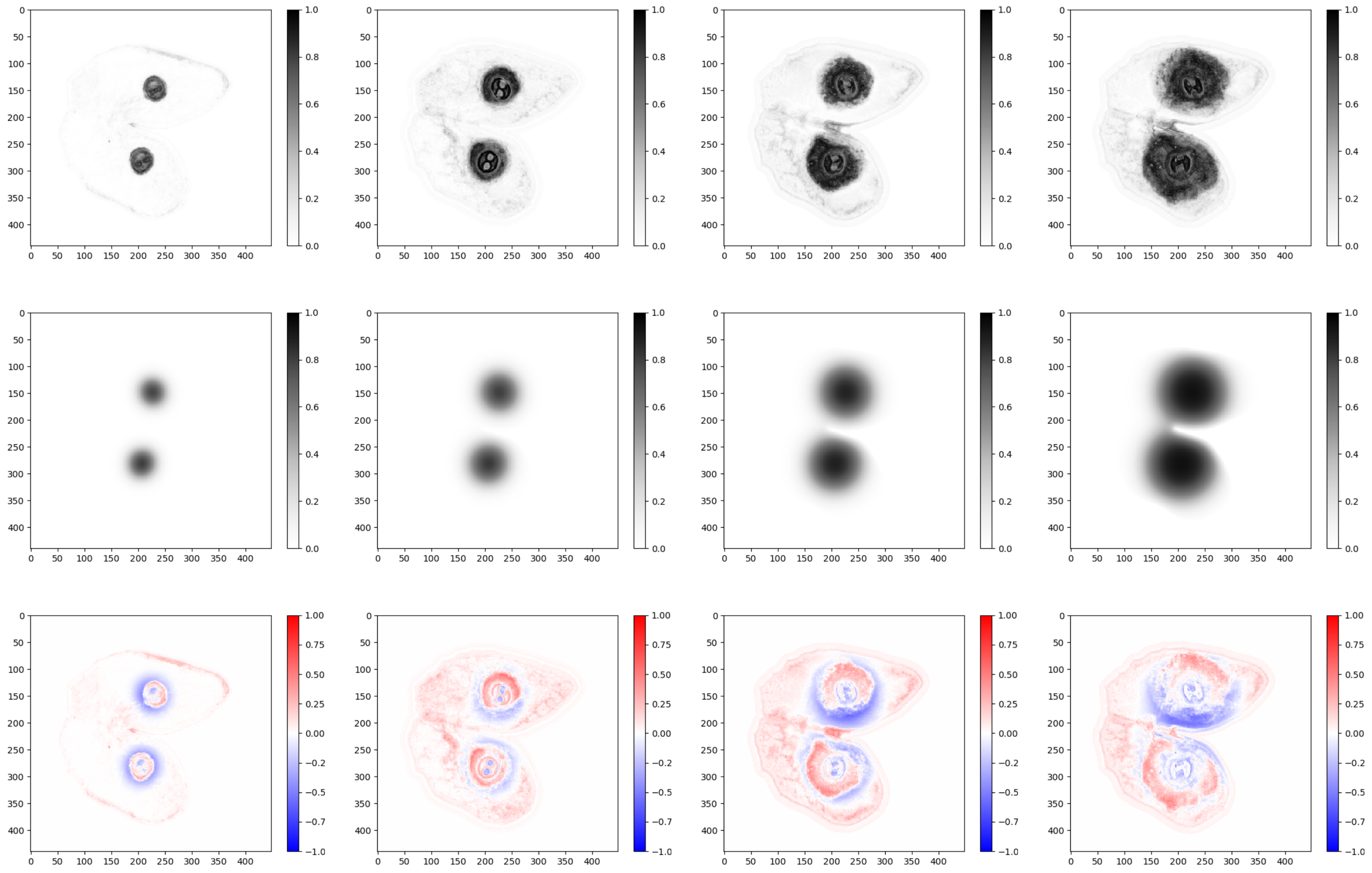

Figure AE: James N°31

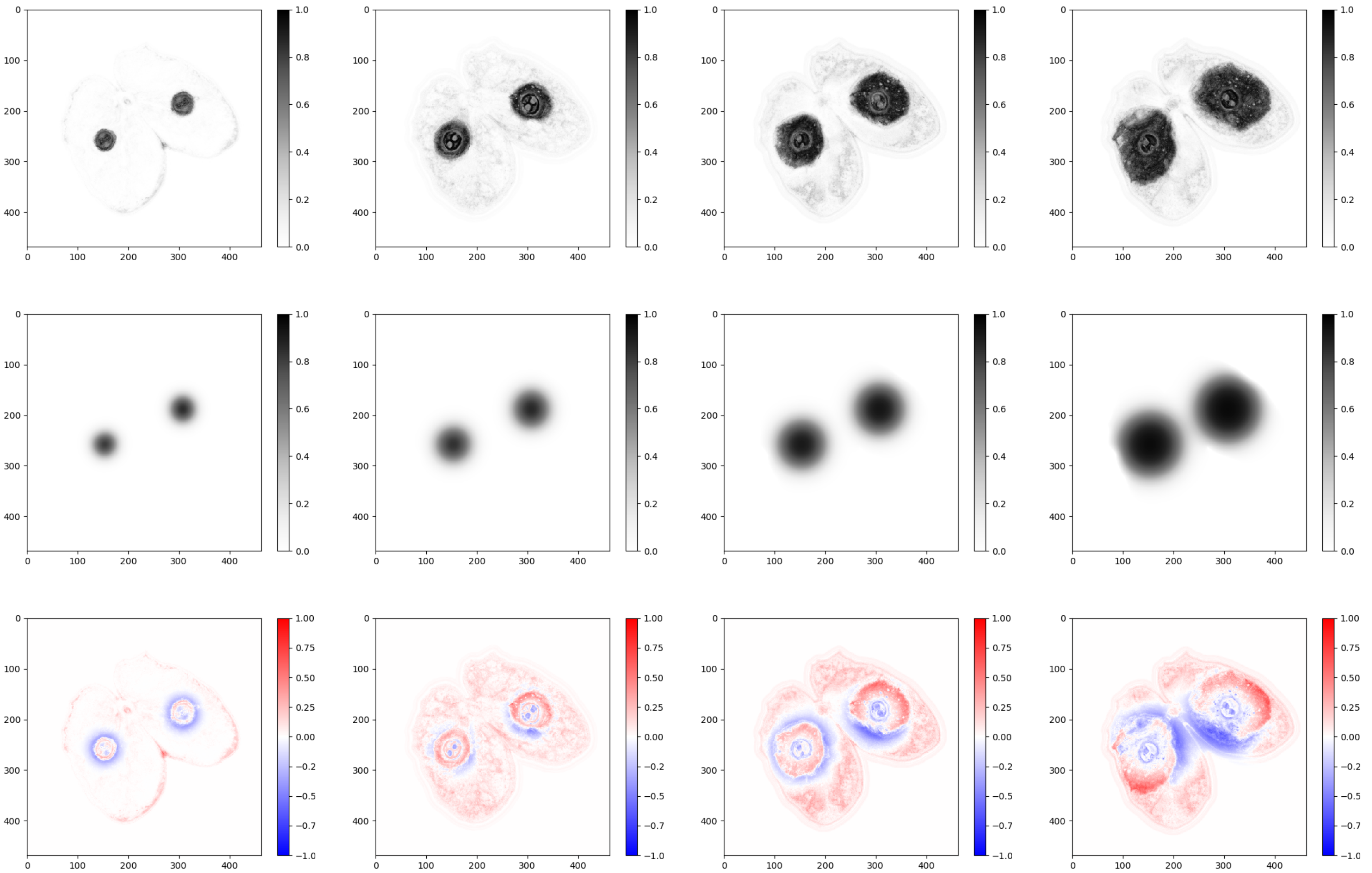

Figure AF: James N°32

S3.2 Visualization of raw residuals

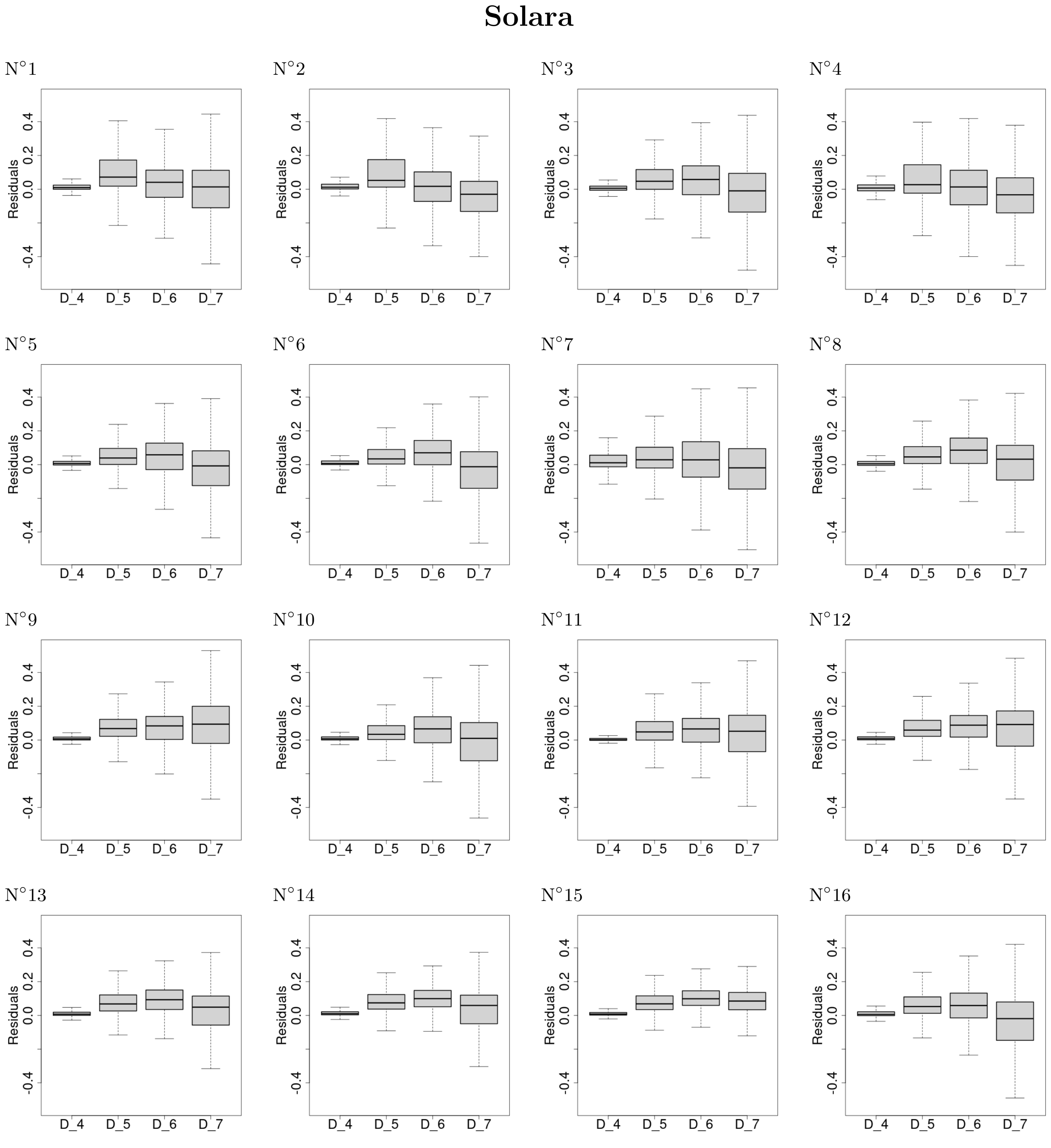

Figure AG: Distribution of raw residuals against dates after inoculation for each Solara stipule pairs.

James

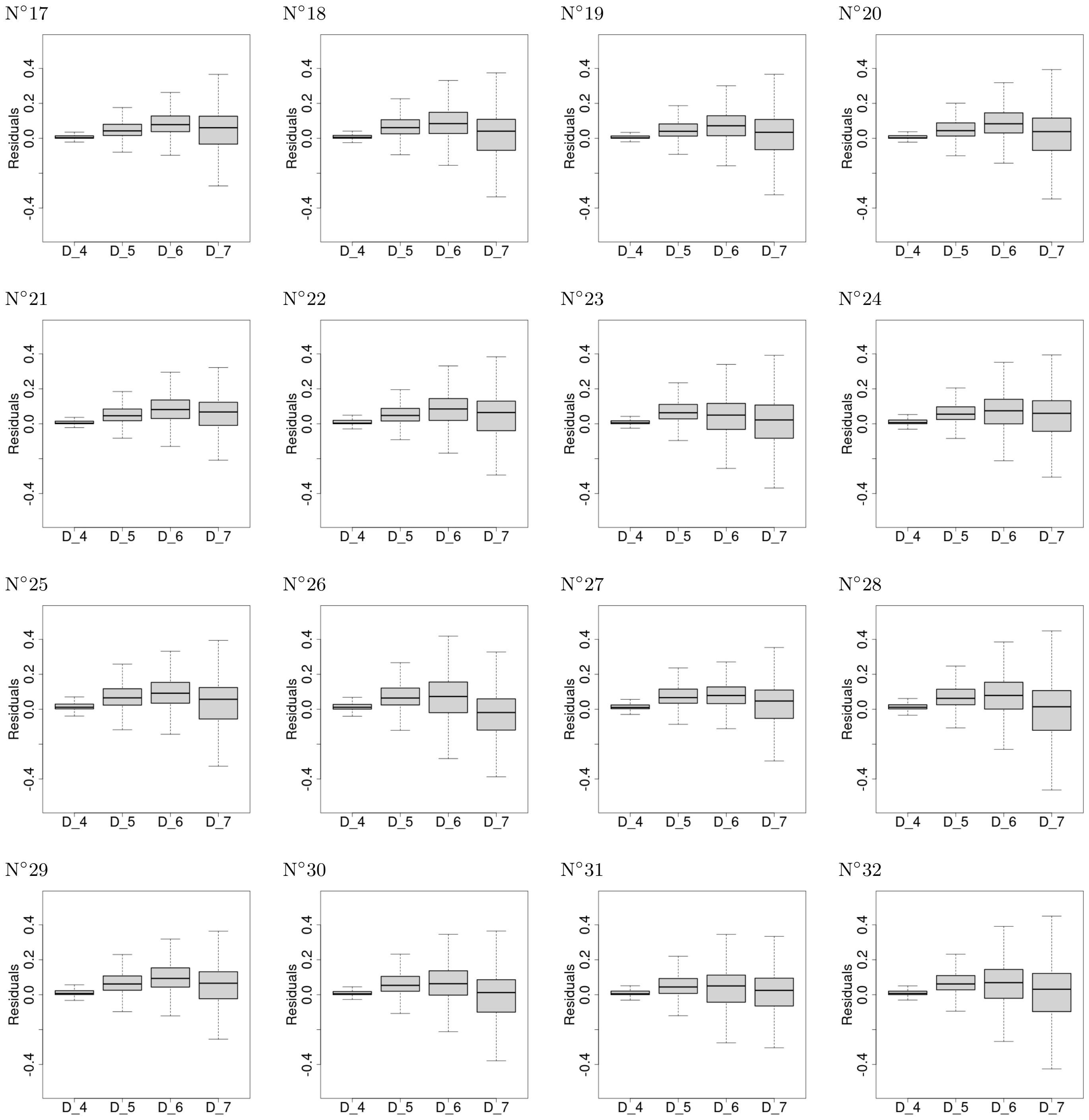

Figure AH: Distribution of raw residuals against dates after inoculation for each James stipule pairs.

S3.3 Model prediction against data (pixel versus pixel)

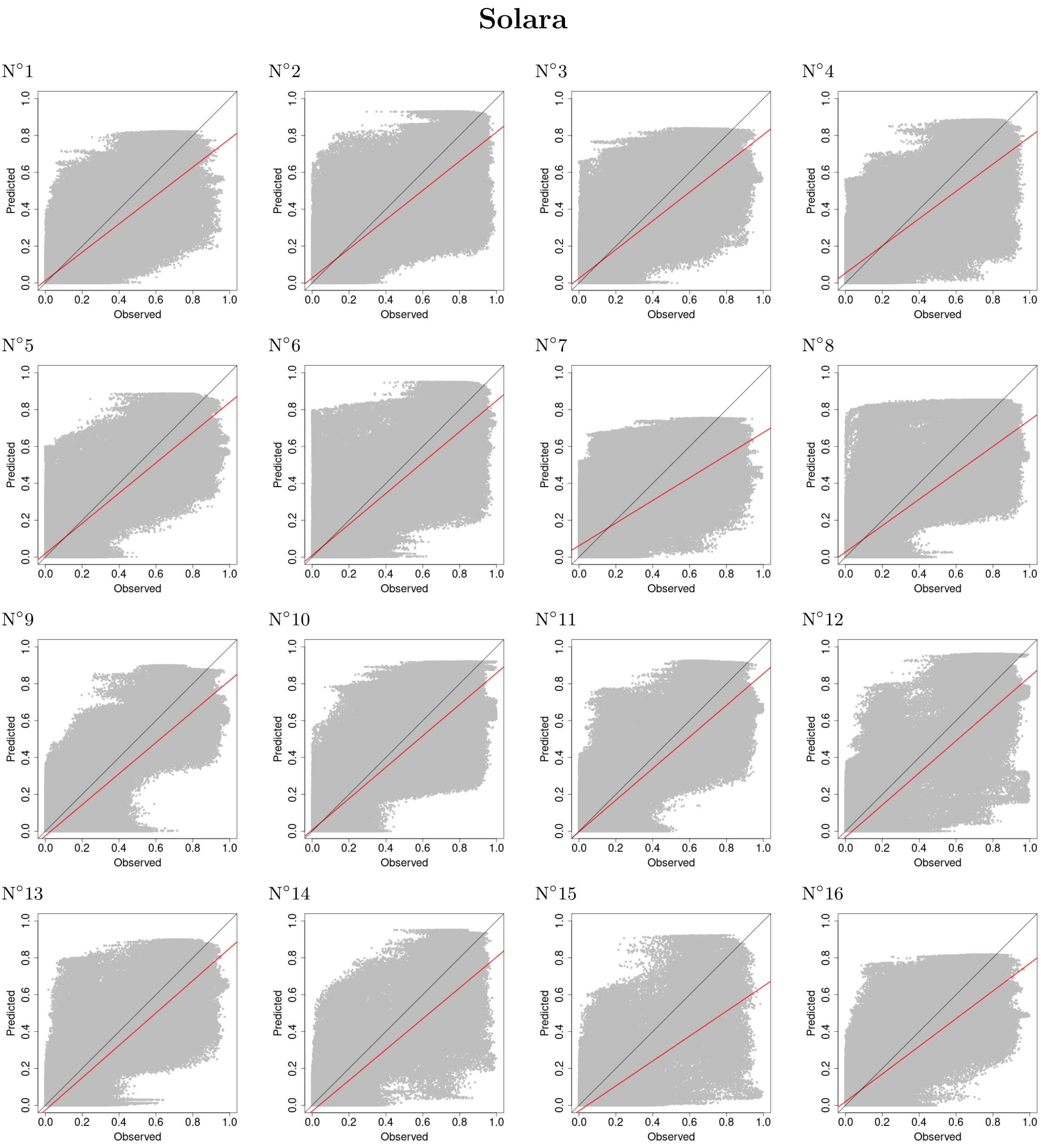

Figure AI: Comparison of infection probability predicted by the reaction-diffusion models against the values of the probability images for the Solara cultivar. The black line is the first bisector that indicates a perfect agreement between values while the red line is the estimated linear relationship between prediction and observation.

James

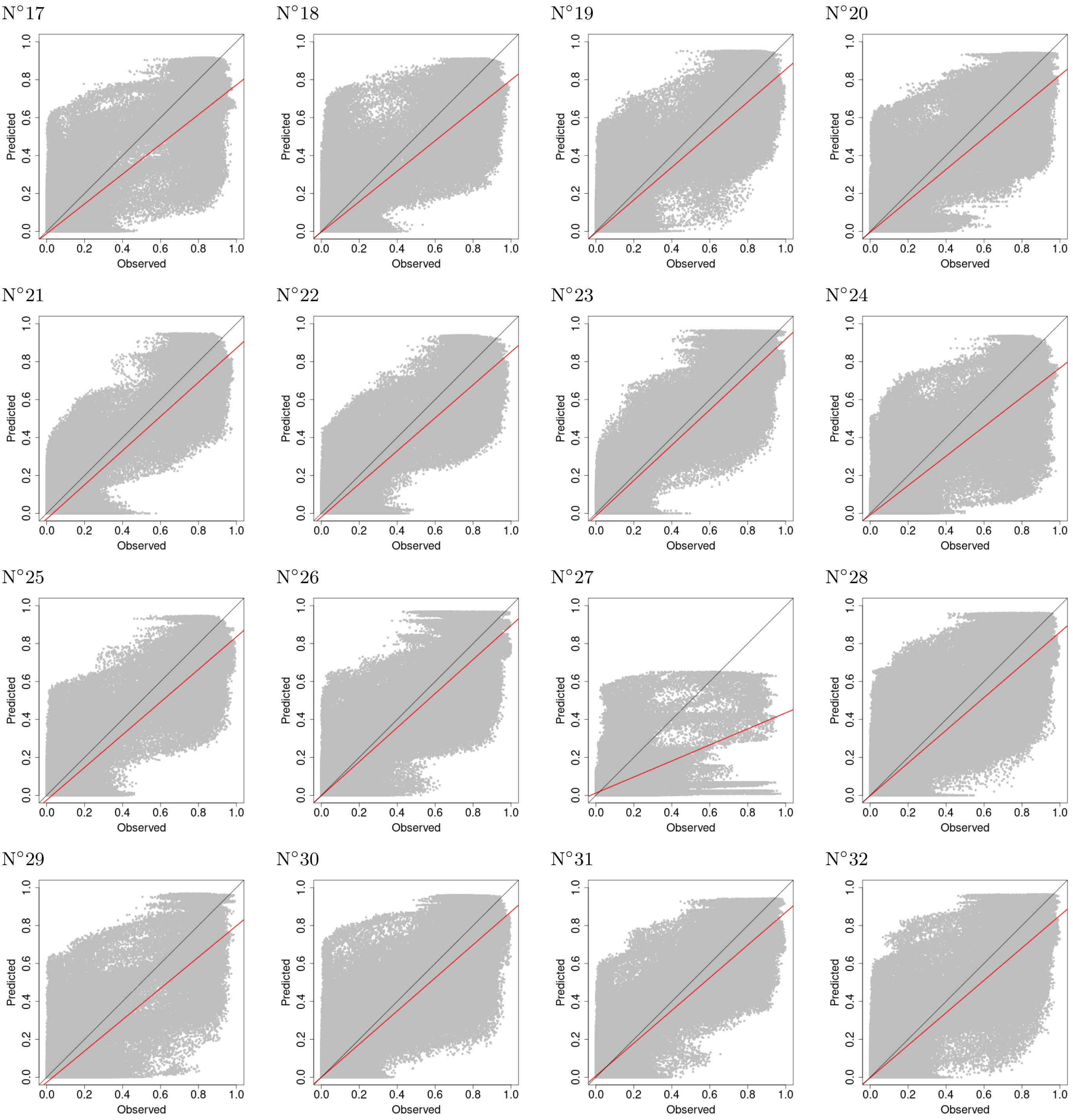

Figure AJ: Comparison of infection probability predicted by the reaction-diffusion models against the values of the probability images for the James cultivar. The black line is the first bisector that indicates a perfect agreement between values while the red line is the estimated linear relationship between prediction and observation.
