# Supplementary material for: Imaging with spatio-temporal modelling to characterize the dynamics of plant-pathogen lesions
Source: PLoS Comput Biol. 2023 Nov 20;19(11):e1011627. doi: 10.1371/journal.pcbi.1011627 (PMC10695395; doi:10.1371/journal.pcbi.1011627)
Supplement: S4 Appendix — (PDF) [file pcbi.1011627.s004.pdf]

Imaging with spatio-temporal modelling to characterize the dynamics of plant-pathogen lesions
Appendix S4

Melen Leclerc[1], Stéphane Jumel[1], Frédéric M. Hamelin[1], Rémi Treilhaud[1], Nicolas Parisey[1], and Youcef Mammeri[2]

[1]IGEPP, INRAE, Institut Agro, University of Rennes, Rennes, France
[2]ICJ, CNRS, Jean Monnet University, Saint-Etienne, France

S4 Numerical discretization and resolution of the optimization problem

S4.1 Meshing the computation domain

The level-set formalism is used to described the stipules surface and boundary. Let ϕ be the level-set function such that the stipules boundary is the zero level of ϕ defined as

$$\partial\Omega := \left\{\mathbf{x} \in \mathbb{R}^2;\ \phi(\mathbf{x}) = 0\right\}.$$

Then the stipules surface is $\Omega := \left\{\mathbf{x} \in \mathbb{R}^2;\ \phi(\mathbf{x}) < 0\right\}$, and its complementary $\Omega^c := \left\{\mathbf{x} \in \mathbb{R}^2;\ \phi(\mathbf{x}) > 0\right\}$. The function ϕ easily provides the exterior normal of leaf as $\vec{n} = \frac{\nabla\phi}{||\nabla\phi||}$. The computation domain consists in a cartesian grid of the overall area (see Figure A). The partial differential equations are solved using explicit Euler finite differences in time and second order centered finite differences in space. The gradient is solved using a descent method [4, 5]. The method is implemented with Petsc using Python [2].

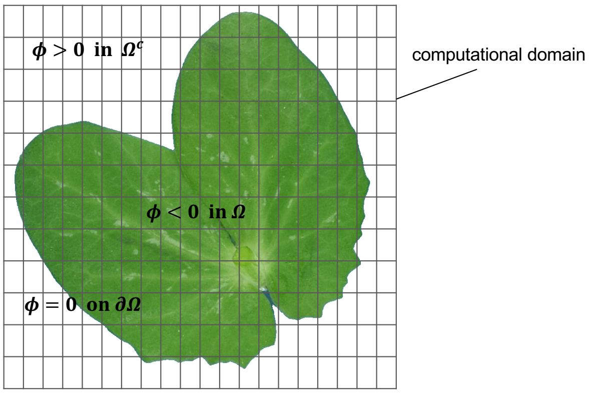

Figure A: Schematic representation of the computational domain given by a cartesian grid and the stipules area $\Omega = \left\{\mathbf{x} \in \mathbb{R}^2;\ \phi(\mathbf{x}) < 0\right\}$ defined by the level-set function ϕ.

S4.2 Variational image data assimilation

The optimization problem is solved thanks to the minimization of the Lagrangian [1]

$$\begin{aligned}\mathcal{L}(a,D) &= \frac{1}{2}\sum_{t=t_3}^{t_7}\int_{\Omega}(u(\mathbf{x},t,\theta)-u_{reg}(\mathbf{x},t))^2 d\mathbf{x} \\ &+ \sum_{t=t_3}^{t_7}\int_{\Omega}\Bigg(\frac{\partial u(\mathbf{x},t,\theta)}{\partial t} - D\Delta u(\mathbf{x},t,\theta) \\ &\qquad - au(\mathbf{x},t,\theta)\left(1-u(\mathbf{x},t,\theta)\right)\Bigg)\lambda(\mathbf{x},t,\theta)d\mathbf{x}.\end{aligned} \quad \text{(Eq A)}$$

The adjoint state λ is the solution of the following backward PDE

$$-\frac{\partial\lambda}{\partial t} = D\Delta\lambda(\mathbf{x},t) + a\left(1-2u(\mathbf{x},t)\right)\lambda(\mathbf{x},t) + \left(u(\mathbf{x},t)-u_{reg}\right) \text{ in } \Omega \quad \text{(Eq B)}$$

with homogeneous Neumann boundary condition $\frac{\partial\lambda}{\partial n}=0$ on $\partial\Omega$, and with the final condition $\lambda(t=t_7)=0$. Finally, the gradient of the cost function is given by

$$\begin{cases}\dfrac{\partial\mathcal{L}}{\partial D} = \displaystyle\sum_{t=t_3}^{t_7}\int_{\Omega}\nabla u(\mathbf{x},t)\nabla\lambda(\mathbf{x},t)d\mathbf{x} \\ \dfrac{\partial\mathcal{L}}{\partial a} = -\displaystyle\sum_{t=t_3}^{t_7}\int_{\Omega}u(\mathbf{x},t)\left(1-u(\mathbf{x},t)\right)\lambda(\mathbf{x},t)d\mathbf{x}.\end{cases} \quad \text{(Eq C)}$$

To avoid local minimum, the initial guess is determined by splitting the Fisher-KPP equation into

$$\frac{du}{dt}(\mathbf{x},t) = au(\mathbf{x},t)\left(1-u(\mathbf{x},t)\right) \quad \text{(Eq D)}$$

$$\frac{\partial u}{\partial t}(\mathbf{x},t) = D\Delta u(\mathbf{x},t). \quad \text{(Eq E)}$$

From the given set of registerd images u_{reg}, the diffusion coefficient in equation (Eq D) is approximated with 98% of the population preservation [3, 6], by

$$D \simeq \frac{\langle r^2\rangle}{16(t_b-t_a)}, \quad \text{(Eq F)}$$

where $\langle r^2\rangle$ denotes the mean of the square radius of mycelium spread between times t_b and t_a. On the other hand, the logistic equation (Eq E) has explicit solution written as

$$u(t_b) = \frac{1}{1+\left(\frac{1}{u(t_a)}-1\right)\exp(-a(t_b-t_a))}$$

that implies

$$a = -\frac{1}{(t_b-t_a)}\ln\left(\frac{\frac{1}{u(t_b)}-1}{\frac{1}{u(t_a)}-1}\right). \quad \text{(Eq G)}$$

The numerical procedure is summarized in Algorithm 1.

Algorithm 1 Parameters identification

Given N registered images u_{reg} and a tolerance τ
Compute the stipules area Ω using level-set
Compute initial D_0 with (Eq F) and a_0 with (Eq G)
while $||\nabla\mathcal{L}(\theta^m)|| \geq \tau$ **do**,
  Find $u^m(\mathbf{x}, t, \theta^m)$ solution of the forward PDE (1)
  Find $\lambda^m(\mathbf{x}, t, \theta^m, u^m)$ solution of the backward PDE (Eq B)
  Compute the gradient

$$\begin{cases} D^{m+1} = D^m - \rho_1^m \left(\sum_{t=t_3}^{t_7} \int_\Omega \nabla u^m(\mathbf{x}, t) \nabla \lambda^m(\mathbf{x}, t) d\mathbf{x} \right) \\ a^{m+1} = a^m + \rho_2^m \left(\sum t = {t_3}^{t_7} \int_\Omega u(\mathbf{x}, t) \left(1 - u(\mathbf{x}, t)\right) \lambda(\mathbf{x}, t) d\mathbf{x} \right) \end{cases}$$

S4.3 Numerical convergence examination

To ensure the convergence of the numerical method, the size of each set of images has been rescaled. Table A indicates that same order values are found for a. The diffusion coefficient depends on images size, but the relative diffusions, *i.e.* the quotient between diffusion coefficient and stipules area are equal at order 10^{-3}. Convergence results are provided in Table A.

	Cultivar						
1	Solara	size	512×514	614×617	717×720	819×822	922×925
		$\hat{a}$	0.4788	0.4835	0.4845	0.4852	0.4841
		$\hat{D}$	0.4597	0.4511	0.4529	0.4534	0.4584
17	James	size	512×514	614×617	717×720	819×822	922×925
		$\hat{a}$	0.4472	0.4492	0.4530	0.4550	0.4543
		$\hat{D}$	0.2367	0.2367	0.2368	0.2366	0.2368

Table A: Estimated diffusion $\hat{D}$, rate growth $\hat{a}$ for two different sets of images with five variable image sizes. The method provides same order parameters with respect to the size images.

References

1 Mark Asch, Marc Bocquet, and Maëlle Nodet. *Data assimilation: methods, algorithms, and applications.* SIAM, 2016.

2 Lisandro D. Dalcin, Rodrigo R. Paz, Pablo A. Kler, and Alejandro Cosimo. Parallel distributed computing using python. *Advances in Water Resources*, 34(9):1124–1139, 2011. New Computational Methods and Software Tools.

3 A. Okubo. *Diffusion and Ecological Problems: Mathematical Models.* Springer-Verlag, Berlin-Heidelberg-New York, 1980.

4 S. Osher and R. Fedkiw. *Level Set Methods and Dynamic Implicit Surfaces.* Springer New York, 2002.

5 J.A. Sethian. *Level Set Methods and Fast Marching Methods: Evolving Interfaces in Computational Geometry, Fluid Mechanics, Computer Vision, and Materials Science.* Cambridge University Press, 1999.

6 N. Shigesada and K. Kawasaki. *Biological Invasions: Theory and Practice.* Oxford University Press, 1997.
