# Supplementary material for: Imaging with spatio-temporal modelling to characterize the dynamics of plant-pathogen lesions
Source: PLoS Comput Biol. 2023 Nov 20;19(11):e1011627. doi: 10.1371/journal.pcbi.1011627 (PMC10695395; doi:10.1371/journal.pcbi.1011627)
Supplement: S5 Appendix — (PDF) [file pcbi.1011627.s005.pdf]

# Imaging with spatio-temporal modelling to characterize the dynamics of plant-pathogen lesions
# Appendix S5

Melen Leclerc[1], Stéphane Jumel[1], Frédéric M. Hamelin[1], Rémi Treilhaud[1], Nicolas Parisey[1], and Youcef Mammeri[2]

[1]IGEPP, INRAE, Institut Agro, University of Rennes, Rennes, France
[2]ICJ, CNRS, Jean Monnet University, Saint-Etienne, France

## S5 Visual assessment of stipules deformation

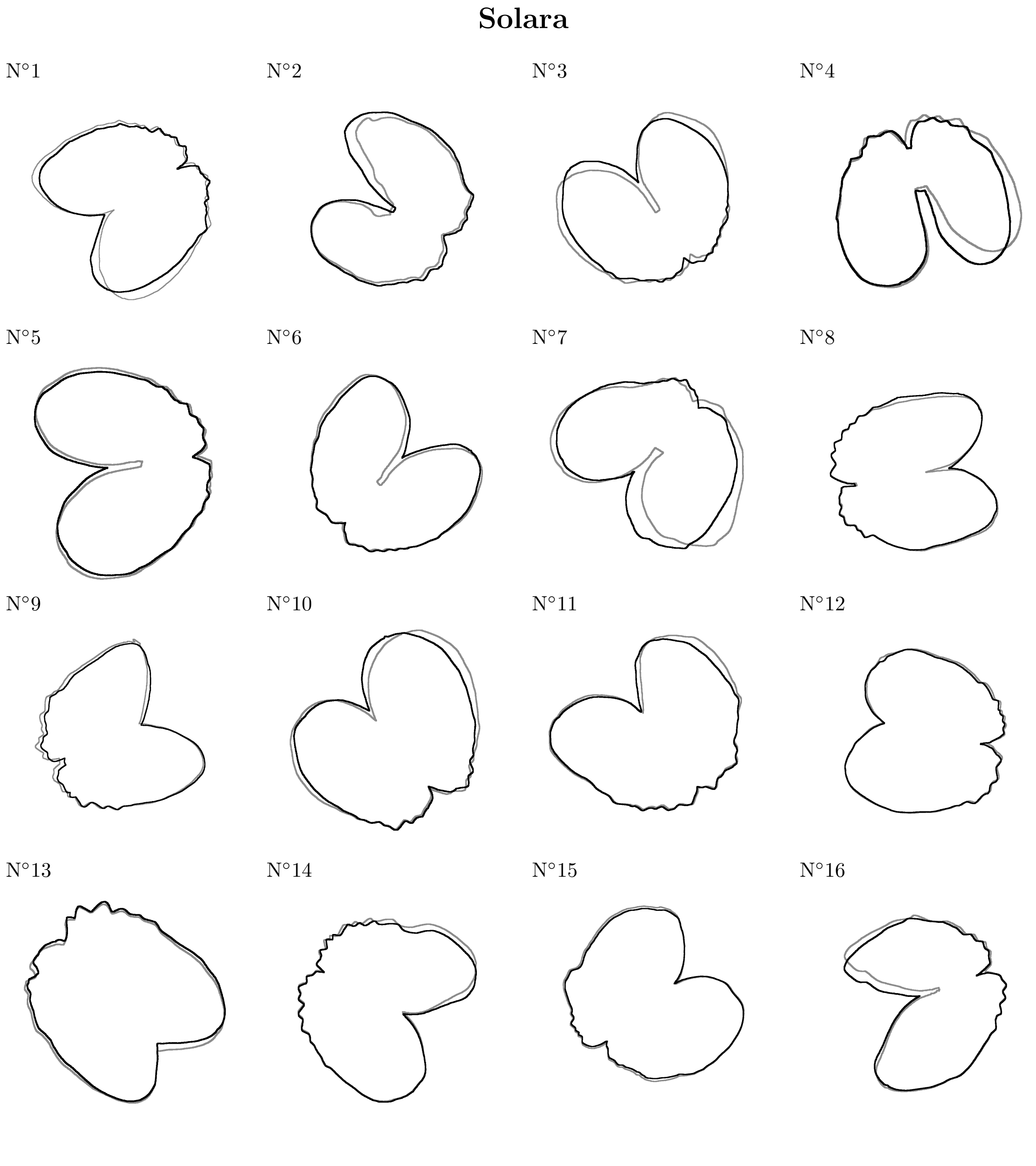


Figure A: Comparison of registered pea stipules for Solara. Stipules edges at day three are in black while those at day seven are in grey.

## James

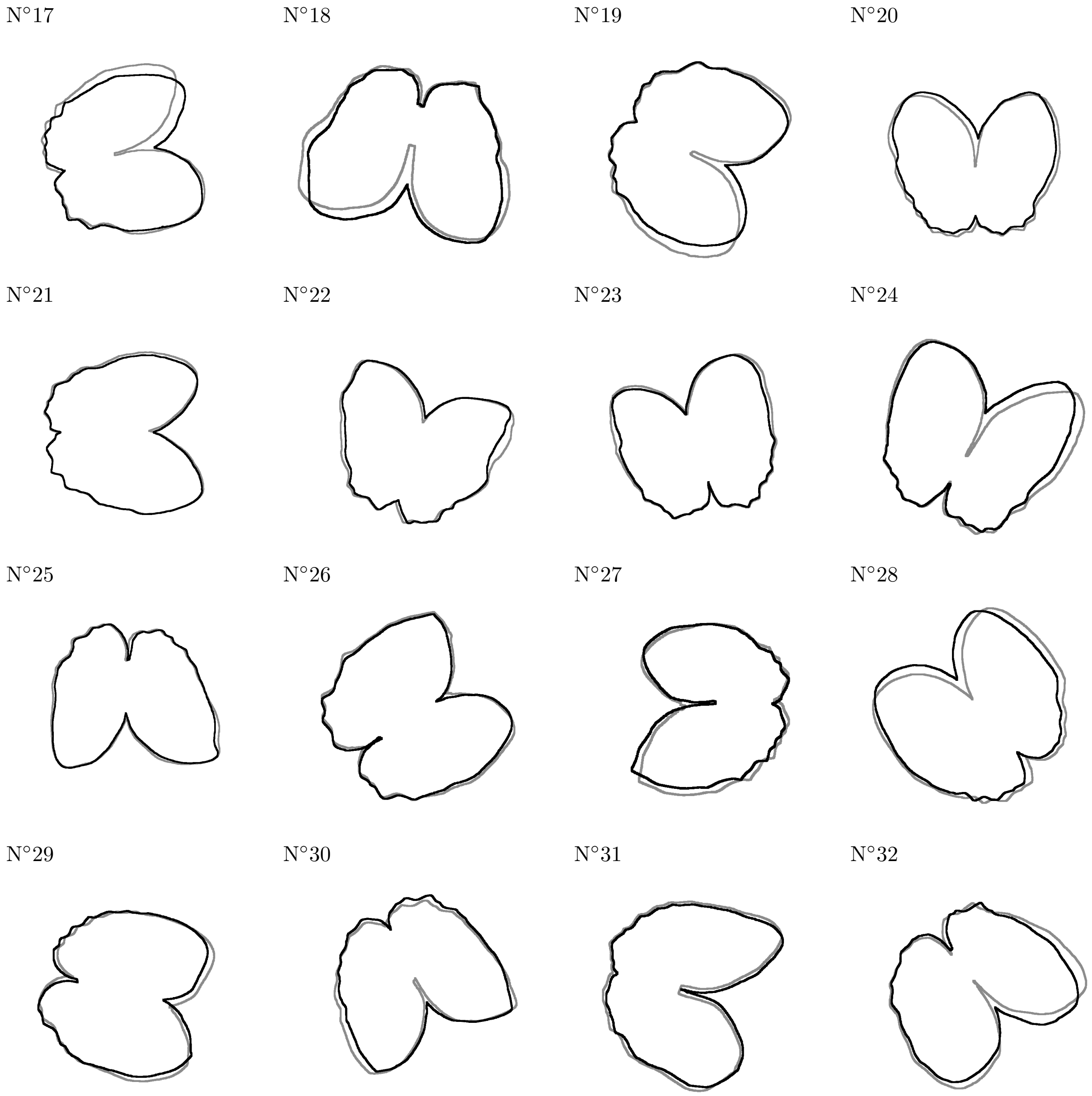


Figure B: Comparison of registered pea stipules for James. Stipules edges at day three are in black while those at day seven are in grey.
