# Supplementary material for: Imaging with spatio-temporal modelling to characterize the dynamics of plant-pathogen lesions
Source: PLoS Comput Biol. 2023 Nov 20;19(11):e1011627. doi: 10.1371/journal.pcbi.1011627 (PMC10695395; doi:10.1371/journal.pcbi.1011627)
Supplement: S6 Appendix — (PDF) [file pcbi.1011627.s006.pdf]

Imaging with spatio-temporal modelling to characterize the dynamics of plant-pathogen lesions
Appendix S6

Melen Leclerc[1], Stéphane Jumel[1], Frédéric M. Hamelin[1], Rémi Treilhaud[1], Nicolas Parisey[1], and Youcef Mammeri[2]

[1]IGEPP, INRAE, Institut Agro, University of Rennes, Rennes, France
[2]ICJ, CNRS, Jean Monnet University, Saint-Etienne, France

S6 Comparison of estimated parameters

S6.1 Estimated parameters

	Cultivar	Leaf Surface (cm^2)	Diffusion $\hat{D}$	Growth rate $\hat{a}$	Cost function J
1	Solara	7.59	4.51e-01	4.84e-01	1.76e-02
2	Solara	6.17	5.49e-01	5.53e-01	1.99e-02
3	Solara	7.41	4.98e-01	5.10e-01	1.65e-02
4	Solara	5.14	5.07e-01	5.24e-01	1.73e-02
5	Solara	7.24	5.34e-01	6.88e-01	1.39e-02
6	Solara	7.10	5.66e-01	5.20e-01	1.93e-02
7	Solara	7.33	4.88e-01	4.61e-01	1.72e-02
8	Solara	8.98	5.85e-01	4.99e-01	1.86e-02
9	Solara	10.10	4.76e-01	4.42e-01	1.52e-02
10	Solara	8.66	6.89e-01	6.02e-01	1.74e-02
11	Solara	8.57	6.84e-01	5.44e-01	1.95e-02
12	Solara	9.32	5.14e-01	5.58e-01	1.54e-02
13	Solara	8.26	3.99e-01	6.37e-01	1.42e-02
14	Solara	8.40	3.04e-01	4.88e-01	1.51e-02
15	Solara	9.99	1.94e-01	5.42e-01	1.25e-02
16	Solara	6.60	3.78e-01	5.23e-01	1.52e-02
17	James	5.21	2.37e-01	4.49e-01	1.01e-02
18	James	3.90	1.83e-01	6.21e-01	8.51e-03
19	James	4.77	3.33e-01	5.16e-01	1.11e-02
20	James	4.99	3.19e-01	4.52e-01	1.22e-02
21	James	5.63	2.70e-01	4.44e-01	9.66e-03
22	James	4.83	2.93e-01	4.35e-01	1.14e-02
23	James	4.18	3.75e-01	4.65e-01	1.22e-02
24	James	4.32	2.60e-01	5.08e-01	1.22e-02
25	James	5.16	2.96e-01	4.73e-01	1.15e-02
26	James	4.54	4.59e-01	5.22e-01	1.51e-02
27	James	2.80	6.69e-02	4.16e-01	7.96e-03
28	James	4.78	4.08e-01	5.30e-01	1.37e-02
29	James	4.28	2.00e-01	6.04e-01	9.92e-03
30	James	4.00	2.87e-01	4.88e-01	1.27e-02
31	James	4.02	3.19e-01	5.00e-01	1.06e-02
32	James	4.39	3.55e-01	5.09e-01	1.15e-02

Table A: Estimated diffusion $\hat{D}$, rate growth $\hat{a}$ and cost function J for 32 different sets of images.

S6.2 Cultivar effect on estimated parameters

		Df	Sum Sq	Mean Sq	F value	Pr(>F)
Diffusion $\hat{D}$						
	Cultivar	1	0.31	0.31	24.95	2.36e-05
	Residuals	30	0.37	0.01		
Growth rate $\hat{a}$						
	Cultivar	1	0.01	0.01	3.56	6.90e-02
	Residuals	30	0.11	0.00		

Table B: Anova table for the cultivar effect on the diffusion $\hat{D}$ and the local growth rate $\hat{a}$. Although the cultivar effect is significant on both $\hat{D}$ and $\hat{a}$ it explains 84% of the variance for the diffusion and only 9% for the growth rate.
