# Supplementary material for: Imaging with spatio-temporal modelling to characterize the dynamics of plant-pathogen lesions
Source: PLoS Comput Biol. 2023 Nov 20;19(11):e1011627. doi: 10.1371/journal.pcbi.1011627 (PMC10695395; doi:10.1371/journal.pcbi.1011627)
Supplement: Attachment — Submitted filename: ReviewPlosComputBiol.pdf [file pcbi.1011627.s009.pdf]

Review of PCOMPBIOL-D-22-01816 « Imaging with spatio-temporal modelling to characterize the dynamics of plant-pathogen lesions »

This is an interesting and timely piece of work that significantly contributes to finely characterize the life history traits involved in the aggressiveness/fitness of plant fungi. As argue by the authors, despite their major interest to understand of plant immunity (and improve disease control), few works have explored how to take advantage of image analysis and mathematical modelling to improve our understanding of plant-pathogen interaction by estimating "hidden" life history traits. This work will significantly contribute to this topic and should likely contribute to renew this field. Plant pathogenic fungi, as most microscopic organisms, offer some specific experimental constraints that might be relaxed by the framework developed by the authors.

It is also remarkable that the authors shared all the necessary material and code in public dataverse, and thus take part in promoting open science.

Major comments

Line 10: "inaccurate traits". We were wondering whether this notion of "inaccurate traits" is related to the notion of "hard traits" and "soft traits" used for example by plant ecologists. Plant ecologists have already emphasized the dilemma between "hard traits", assumed to capture the function of interest, and "soft traits", more indirectly linked with performance but more easily measurable (e.g. Violle C. et al. 2007. Oikos 116 : 882-892). If so, these links should deserve to be mentioned.

Lines 47-50: Although concerning the form of the article, the actual presentation of the results is not satisfactory, and does not follow the plan announced in the introduction (lines 47-50). In its current form, the material and methods section should be read before the results section. The content of the material and methods and results sections should be adapted to the format recommended by Plos Computational Biology while allowing an understanding of the work carried out and the approach adopted by the authors (e.g. : the need to align the lesions, line 75). A minor linked point is that supplementary materials should be listed in the order they appear in the text (e.g. line 62).

Equation 2: How longitudinal data (i.e. repeated measures of the same individuals in time, leading to non-independence between observations) are taken into account in the model inference procedure? Similarly, non-independence between observation can rise for close pixels in the images at a given time. We understand from equation 2 that the authors assumed that the observations are independent, is this the case? Ignoring these sources of spatial and temporal dependence can bias the estimated parameters (mean value and/or confident interval). In the worst case it could impact the conclusion of the paper regarding the traits differences between the two cultivars James and Solara. The author could address this point or at least discuss it in a dedicated paragraph of the manuscript.

Additional point: Why the authors did not used a model including latency time as an additional parameter, in particular since this is a trait classically considered for plant fungi (see line 5/6)? It would allow to use the images starting from the inoculation time (instead of from day 3). Related to this, are the lesion appearing at day 3 (t0 see line 290) in all images? Or are there some variabilities in the day of apparition of symptoms? More generally, can the author discuss the use of model explicitly including the age of infection as an (additional) structuring variable in their PDE model (as in

reference 38). Such improvement could help to identify traits such as the spore production function (which typically depends on the age of infection/lesion), or the effect of ontogenetic and disease-induced change in host susceptibility as cited line 170.

Minor comments

Line 3 to 6 : maybe, given the journal, adding a definition of “life-history traits” and the way they relate to fitness (a more familiar notion) should be useful.

Line 3 : change “determine” by “identify” ?

Lines 4-5 : the list of traits given here (as well as Ref 1 and 2) is more specific of plant fungi, while the authors here refer to pathogen (which also include virus, bacteria for which others traits are measured). The authors need to clarify the context better, or speak more generally and explicitly

Line 29 : Another classical reference of such work is Gilchrist et al, 2006, Evolution, 60(5), 970-979.

Lines 80-81 : it seems from the formulation adopted that the parameters *a* and *D* refer to the cultivar, while they characterize the propagation of the pathogen in the two cultivars used. A reformulation should help here (e.g. see lines 100-101).

Line 170: the appropriate term is maybe “ontogenic” (eg. Ficke et al, 2002, Phytopatlogy, 92(6), 671-675) rather than ontogenetic. To be check.

Line 64-66: It could be interesting to provide another metric of model fit, such as a plot comparing observed and fitted values (for each day from 4 to 7) of the proportion of symptomatic area. Such a metric will be a simpler way to evaluate the quality of the model fit.

Lines 225: It is not clear why some pixels can be classified in “background” state , as it seems that the stipule images have already been extracted from raw images (line 220).

Lines 236: Why the authors define a cut-off probability of 0.5 with 3 possible states (healthy, symptomatic and background)? Is there any reference supporting the choice of this probability? In particular, it raises question about how pixels with no probability above 0.5 are handle in the described process (for example probabilities of 0.4, 0.4 and 0.3 respectively for a pixel to be healthy, symptomatic and background). More generally, in the overall process, we did not get how this classification in three states is used, as probability images rely on continuous measures of probability (see Fig 4C).

Line 295. Why the authors have preferred an anova strategy to compare the estimated parameters on each cultivar (line 82) rather than more classical metrics (eg. AIC with models fitted to all the dataset and considered from 2 to 4 parameters of interest?)

SI3: The figure is labelled as S1 (instead of S3) and refers to Fig. 1c instead of, presumably Fig. 4c

SI5: It in not clear what are the grey vertical lines in the boxplots. Moreover, it would be useful to indicate the range of the number of points (we guess pixels) used to draw each boxplot.

Figure 2 : add a title (and unit) on the x-axis. Moreover, the Jaccard Index is expressed between 0-1, while in the text is presented as a percentage (line 238). Note that at line 74, when talking about Jaccar index, a percentage is missing (it should be 80%).

Additional point: It should be interesting to compare the traits *a* and *D* estimated in the present work to the traits classically estimated on this same pathosystem (references 17 and 20). The authors could comment these differences and possibly further argue on the adding value of their framework.
