# Supplementary material for: Imaging with spatio-temporal modelling to characterize the dynamics of plant-pathogen lesions
Source: PLoS Comput Biol. 2023 Nov 20;19(11):e1011627. doi: 10.1371/journal.pcbi.1011627 (PMC10695395; doi:10.1371/journal.pcbi.1011627)
Supplement: Attachment — Submitted filename: ReviewPlosComputBiol_turn2.pdf [file pcbi.1011627.s011.pdf]

Review of PCOMPBIOL-D-22-01816 « Imaging with spatio-temporal modelling to characterize the dynamics of plant-pathogen lesions »

The authors did a major effort to answer to our questions and to integrate our comments. In our view this new version of the paper is much more easy to follow and understand, giving some important precisions to the points raised by the reviewers. However, we still have some minor comments.

1) We previously showed our concern about the spatial and temporal correlation in pixel data. In the Material and Methods section of the new version of the MS, the authors highlighted that they neglected this correlation. However, contrarily to our suggestion, they did not discuss the implication of this strong assumption. We still believe that at least the nature of the possible bias in parameter estimates (mean value and/or confident interval) generated by this assumption should be mentioned and discussed as a possible extension/improvement of the method. The reader could ask themselves whether these possible biases are negligible with dataset including tens of thousands of pixels. This is why we suggest to more deeply discuss this issue.
2) The authors updated the text at lines 91-116 for describing image processing. However, regarding one of our previous comment, it is still not clear why some pixels can be classified as “background” at step 3, after having applied the SIOE algorithm, which already extracted the stipules from the image, at step 1. Is the “background” at line 94 different from the “background” at line 98?
3) Although we get the sense of lines 179-183, we think that the computation of the lesion size from probability maps is not easy to understand. We suggest to explain this point in two steps. Firstly, the authors estimated the observed (respectively estimated) lesion size by counting the number of pixel with probabilities higher than 0.5 from probability maps (respectively model outputs), for each image. Secondly, the authors compare these observed and estimate values in Fig. 4. Another question here is whether the authors considered the 32 images (16 images X 2 cultivars) or the 64 images (16 images X 2 stipules X 2 cultivars)? Another question is whether the lesion size was estimated only at day 7th? Finally, the author could consider to distinguish the points in Fig.4 for James and Solara cultivars with different shapes.
4) Line 223 the values of coefficient are (likely) inverted. They should be:
“*The spread of the pathogen on James cultivar was characterized by mean growth rate a and diffusion coefficient D of respectively 1.33 and 0.55 against 1.26 and 1.54 on the more susceptible Solara*.”
5) At line 224 and Fig.5, the authors should mention (if we understood correctly) that the distributions are based on the 32 values estimated for each cultivar.
6) Appendix 3: why not jittering the outliers in figures S32-34? Moreover, the authors should improve figures’ S1-S31 axes in the appendix 3, they are really difficult to read.

In addition to our previous comments, we would like to raise few additional points:

1) For the readers of PLoS CB, it could be interesting that the authors shortly explain why “it is more relevant to rely on data assimilation method (line 151-152)”.

2) To avoid repetitions from the *Image processing* section, the authors could consider to rephrase lines 159-163 with only: "*In our cases the probability images (Figure 1) were considered as the observation $u_{reg}(x,t)$ in the cost function.*"
3) Figure 2: "stipules of Solara (a) AND (not ET) James (b)". Also, the authors should mention in the legend that "1" and "17" are plant numbers.
4) At line 120: how is the fit quality of non-spatial model?
5) At line 125, replace with "appearance of symptomatic host tissue" ? (as the model does not consider leaf growth).
6) At line 53, 117 (and in many other parts throughout the paper), the authors state that the model describes the spread of necrotic lesion on host tissue, which is effectively the case. We wonder whether defining *u* as the "probability that a host is infected" (at line 131), is not somewhat misleading. The author could say that *u* is the probability of a host at location x at time t to be necrotic as the result of the final step infection process.
