# Supplementary material for: Imaging with spatio-temporal modelling to characterize the dynamics of plant-pathogen lesions
Source: PLoS Comput Biol. 2023 Nov 20;19(11):e1011627. doi: 10.1371/journal.pcbi.1011627 (PMC10695395; doi:10.1371/journal.pcbi.1011627)
Supplement: Attachment — Submitted filename: Response_to_reviewers.docx [file pcbi.1011627.s010.docx]

Dear Editor,

Thank you for your having arranged such useful reviews of our article “Imaging with spatio-temporal modelling to characterize the dynamics of plant-pathogen lesions” submitted to PloS Computational Biology. We thank the reviewers for their constructive comments that were useful for improving the initial version of the manuscript and clarify some important points. We have revised the paper in line with their suggestions. In particular, we have re-estimated all the parameters with an improved version of the optimization aglorithm (adaptive learning rate in the gradient descent) and most importantly we have performed a more detailed comparison between fitted models and data that greatly improved the presentation of our work. The revisions and the answer to reviewers are addressed below.

Thank you for considering our article,

Yours sincerely,

Melen Leclerc

On behalf of the co-authors: Stéphane Jumel, Frédéric Hamelin, Rémi Treilhaud, Nicolas Parisey & Youcef Mammeri

Reviewer #1:

The manuscript presents a study where the expansion of a disease lesion in a leaf was observed using imaging and image analysis, and subsequently the expansion was modelled with Fisher-KPP model that explicitly includes local growth and diffusion to new area. The main biological claim is that partial resistance in a cultivar (in this particular case) is more governed by hindering invasion (diffusion) than local growth. However, the main scientific advance is the use of temporal imaging and mechanistic modelling to study growth of individual lesions (i.e. the methods), while generalizable knowledge about invasion vs local growth would require more work.

In my opinion this approach (temporal imaging and mechanistic modelling) is generally very welcome and will eventually improve our understanding of within host dynamics significantly.

However, I have hard time believing the suitability of the chosen model to understand the lesion growth dynamics. It is hard to evaluate how well the model really corresponds to the data. Based on the videos and snapshot images, it is clear that the model predicts circular probability-of-infection contours, where in practice part of the contour area is certainly infected and part is certainly healthy - resulting in moderate probability of infection. It seems to me, that the model misses the actual lesion expansion dynamics. Having 60-degree angle where the lesion has expanded and tissue became clearly symptomatic, and 300 degrees of clearly healthy, is not well captured by circular contour of 16.7% infection probability. Hence, I'm not convinced of the biological relevance of the results. I believe that a model that allows for anisotropic expansion is needed for gaining biological understanding of the processes of lesion expansion. Higher time resolution would also potentially make it more convincing - now the dynamics is based on four snap shots and the lesion expansion between each of those is quite significant.

We thank the reviewer for their feedback. We performed a more detailed comparison between fitted models and data (that are actually probability images given by trained segmentation classifiers). These analyses show some discrepancies between the Fisher-KPP model and the image data as the reaction-diffusion model tends to underestimate infection probability. Despite these observed errors, that can be partially explained by the increasing difficulty to segment visible lesions with time, the lesion surface predicted by the model (after applying a 0,5 threshold on the infection probability) is well correlated with the one obtained through pixel-based segmentation. Therefore, even if some hypothese made in this article could be relaxed to even better describe lesions we believe that our model capture the essential patterns of the data and provides a better understanding of lesion spread than that is usually done in phytopathology with lesion size phenotypic data and temporal models (when used at all). Considering only temporal data it is common to get errors and discrepancy between models and data. Yet when it can be fitted to the data, a mechanistic model offers a mean to gain knowledge on the main epidemiological processes. When data are images the errors can perhaps appear less acceptable as such data remain seldom considered by modellers. However, fitting parsimonious models to these data captures the main patterns and already gives original information.

The idea of using videos would probably help to better describe lesions growth. However in this case it would be more relevant to consider other mathematical models that would capture better lesions contours (e.g. Level-Set methods for computer vision). Nevertheless some of these models could « overfit » the data and provide less mechanistic knowledge. Moreover, as the parameters of the Fisher-KPP equations are theoretically identifiable with two images (see Cristofol & Roques 203, ref 35 ) there is no need to accumulate large data set to fit simple spatial models.

In addition to this answer, we hope that the new results as well as the changes we made to the manuscript will convice the reviewer.

**Action :** As explained lines 176-183 in the M&M we compared models’ predictions with image-data. For all stipules and dates used in parameters estimations we included in Appendix S3 images of fitted model prediction, probability images (data) and images of raw resiiduals. Moreover, for each stipule we compared the values of predicted pixels against values in the probability images (data) by plotting the data and estimating the linear relationship (predicted ~ data). All these figures are available in Appendix S3. In addition, as proposed by reviewer 2 we compared the lesion size (in pixels) predicted by the model, by thresholding the infection probability, with the one given by the pixel-based segmentation. The results are given in a new figure in the main text (i.e. Figure 4) that shows the good correlation between model and data. The result part has thus been changed to present these new findings (lines 192-218).

This conclusion is really unfortunate, since I think the idea is really valuable, and definitely something that will lead to deeper understanding of pathogen dynamics. Perhaps the idea on its own already merits publication, but here the models correspondence to biology is unfortunately not convincing to me. I would be happy to be proven wrong.

We agree that more general conclusion regarding the effect of quantitative host resistance on diffusion and local growth would require more experimental results on a larger panel of plant genotypes/cultivars. We believe that our study of only two cultivars with contrasted levels of resistance rather illustrate the potential of combining imaging with mathematical modelling to improve our knowledge on plant-pathogen interactions. We thus agree that the originality of this work is more the methodology than the biological results. The new version of the manuscript may hopefully prevent such misunderstanding.

**Action :** To prevent misunderstanding regarding the generecity our biological results and to rather emphasize the potential of using such original methods in plant pathology we modified the text in the summary, the introduction (line s57 – 60), the results (lines 227 – 230), and the discussion (lines 256 – 259, 265 – 268, 272 – 274, 295 – 298).

Minor comments:

LL 285-286: Are there examples of this in literature?

Such approach is actually common in biomedical sciences when fitting tumor growth model to image data. (see Hogea et al. 2008 in our references, ref 8)

LL 292-293: Are stipules area the same as image size, i.e. is it the image size after extracting the stipules from the background? This could be clarified.

Stipules area were obtained on the raw images using a dedicated landmarks in the acquisition setup. We included the information in lines 172 – 173.

LL 296-297: Refer to S5 Appendix where you show the residuals.

We included the reference, thank you.

S3 figure: probability plot would be likely more informative as 2D where just the colors are used for showing probability (Excluding height) - especially since it seems to be discrete (or discretized?)

As all probability images are now availble in Appendix S3 we removed this figure.

S7 appendix, Anova table S2: "Although the cultivar effect is significant on both D and a it explains 84% of the variance for the diffusion and only 9% for the growth rate." - the p-value is 0.069 for parameter a, which makes the above claim a bit unconventional, although we should not be stuck with a godly threshold of p=0.05. This might be good to note, if some readers think that only p<0.05 is significant.

We agree with the reviewer and changed to more conventional interpretation of p-value. Moreover, the new results with improved learning parameters used in the gradient-descent no-longer suggest a significant difference (even at a higher threshold than 0.05) of growth rate (see Figure 5 and Appendix S8).

Reviewer #2:

This is an interesting and timely piece of work that significantly contributes to finely characterize the life history traits involved in the aggressiveness/fitness of plant fungi. As argue by the authors, despite their major interest to understand of plant immunity (and improve disease control), few works have explored how to take advantage of image analysis and mathematical modelling to improve our understanding of plant-pathogen interaction by estimating “hidden” life history traits. This work will significantly contribute to this topic and should likely contribute to renew this field. Plant pathogenic fungi, as most microscopic organisms, offer some specific experimental constraints that might be relaxed by the framework developed by the authors. It is also remarkable that the authors shared all the necessary material and code in public dataverse, and thus take part in promoting open science.

We thank the reviewer for his very positive comments on the initial version of the manuscript.

Major comments

Line 10: “inaccurate traits”. We were wondering whether this notion of “inaccurate traits” is related to the notion of “hard traits” and “soft traits” used for example by plant ecologists. Plant ecologists have already emphasized the dilemma between “hard traits”, assumed to capture the function of interest, and “soft traits”, more indirectly linked with performance but more easily measurable (e.g. Violle C. et al. 2007. Oikos 116 : 882-892). If so, these links should deserve to be mentioned.

After having checked the notion of hard/soft traits we agree with the reviewer that traits obtained through mechanistic models could be considered as hard traits while common « inaccurate » may be soft traits. As plant pathologist are perhaps less familiar with this concept we followed the suggestion made by the reviewer and mentioned it in the discussion.

**Action :** We included the suggested reference (now ref 39) and discussed the dychotomy of soft/hard traits in the discussion (260 – 265).

Lines 47-50: Although concerning the form of the article, the actual presentation of the results is not satisfactory, and does not follow the plan announced in the introduction (lines 47-50). In its current form, the material and methods section should be read before the results section. The content of the material and methods and results sections should be adapted to the format recommended by Plos Computational Biology while allowing an understanding of the work carried out and the approach adopted by the authors (e.g. : the need to align the lesions, line 75). A minor linked point is that supplementary materials should be listed in the order they appear in the text (e.g. line 62).

Thank you for raising this point.

**Action :** As the materials and methods are central for this work and PloS CB allows to include M&M before the result section we did so. We belive that this new order is more suitable for this work. We also modified the number of appendices according to their order of appearance in the text.

Equation 2: How longitudinal data (i.e. repeated measures of the same individuals in time, leading to non-independence between observations) are taken into account in the model inference procedure? Similarly, non-independence between observation can rise for close pixels in the images at a given time. We understand from equation 2 that the authors assumed that the observations are independent, is this the case? Ignoring these sources of spatial and temporal dependence can bias the estimated parameters (mean value and/or confident interval). In the worst case it could impact the conclusion of the paper regarding the traits differences between the two cultivars James and Solara. The author could address this point or at least discuss it in a dedicated paragraph of the manuscript.

As pixel data in image sequences are highly spatially and temporally correlated, we agree that it would be worth integrating the spatio-temporal correlation of observation errors in the cost function. However, in practice it is still difficult to estimate covariance and cross-covariance matrices in image-data assimilation and ignoring the error strucutre remains common.

**Action :** We mentioned this issue and added a reference (*Chabot, V., Nodet, M., Papadakis, N., & Vidard, A. (2015). Accounting for observation errors in image data assimilation. Tellus A: Dynamic Meteorology and Oceanography, 67(1), 23629., ref 36*) in lines 167 – 170.

Additional point: Why the authors did not used a model including latency time as an additional parameter, in particular since this is a trait classically considered for plant fungi (see line 5/6)? It would allow to use the images starting from the inoculation time (instead of from day 3). Related to this, are the lesion appearing at day 3 (t0 see line 290) in all images? Or are there some variabilities in the day of apparition of symptoms? More generally, can the author discuss the use of model explicitly including the age of infection as an (additional) structuring variable in their PDE model (as in reference 38). Such improvement could help to identify traits such as the spore production function (which typically depends on the age of infection/lesion), or the effect of ontogenetic and disease- induced change in host susceptibility as cited line 170.

Estimating the intial conditions would indeed be very interesting as it is difficult to monitor the location of initial symptoms that appears in the drop used for inoculation. While this is theoretically doable with the same modelling framework we haven’t tried yet. This is particularly important because the initial conditons are knwon to influence the propagation of KPP systems (Hamel, F., & Roques, L. (2010). Fast propagation for KPP equations with slowly decaying initial conditions. *Journal of Differential Equations*, *249*(7), 1726-1745.). Using age-structured PDE to take into account the change in host susceptibility is extacly what we have in mind for future work.

**Action :** We mentioned the possibility of considering such PDE model in the discussion (lines 322 - 323) and included a relevant reference that uses age-structured PDE model for the propagation of plant diseases (*Burie, J. B., Ducrot, A., & Mbengue, A. A. (2017). ASYMPTOTIC BEHAVIOUR OF AN AGE AND INFECTION AGE STRUCTURED MODEL FOR THE PROPAGATION OF FUNGAL DISEASES IN PLANTS. Discrete & Continuous Dynamical Systems-Series B, 22(7). ref 54*)

Minor

Line 3 to 6 : maybe, given the journal, adding a definition of “life-history traits” and the way they relate to fitness (a more familiar notion) should be useful. Line 3 : change “determine” by “identify” ?

Lines 4-5 : the list of traits given here (as well as Ref 1 and 2) is more specific of plant fungi, while the authors here refer to pathogen (which also include virus, bacteria for which others traits are measured). The authors need to clarify the context better, or speak more generally and explicitly

Line 29 : Another classical reference of such work is Gilchrist et al, 2006, Evolution, 60(5), 970-979.

We thank the reviewer for these relevant suggestions.

**Action :** We modified the first lines of the introduction (lines 2- 8) to clarify these points and included the reference in the manuscript (ref 20).

Lines 80-81 : it seems from the formulation adopted that the parameters a and D refer to the cultivar, while they characterize the propagation of the pathogen in the two cultivars used. A reformulation should help here (e.g. see lines 100-101).

Sorry for this langage abuse.

**Action :** We modified the mentioned lines to clarify the message (now lines 221 - 223).

Line 170: the appropriate term is maybe “ontogenic” (eg. Ficke et al, 2002, Phytopatlogy, 92(6), 671- 675) rather than ontogenetic. To be check.

After having checked it appears that both terms are used (e.g. West, G. B., Brown, J. H., & Enquist, B. J. (2001). A general model for ontogenetic growth. *Nature*, *413*(6856), 628-631.).

Line 64-66: It could be interesting to provide another metric of model fit, such as a plot comparing observed and fitted values (for each day from 4 to 7) of the proportion of symptomatic area. Such a metric will be a simpler way to evaluate the quality of the model fit.

Fthank you for this comment and suggestion. Indeed, a more detailed analysis of the goodness of fit was lacking in our initial manuscript.

**Action : F**ollowing reviewer1’s concern we performed a detailed assessment of the goodness of fit. The predicted symptomatic surface was actually a good metric as, despite some discrepancy between infection probability given by the model and the classifiers, it appeared that the KPP model described well enough the lesions surface. The new results are given in figure 4 and Appendix S3.

Lines 225: It is not clear why some pixels can be classified in “background” state , as it seems that the stipule images have already been extracted from raw images (line 220).

Lines 236: Why the authors define a cut-off probability of 0.5 with 3 possible states (healthy, symptomatic and background)? Is there any reference supporting the choice of this probability? In particular, it raises question about how pixels with no probability above 0.5 are handle in the described process (for example probabilities of 0.4, 0.4 and 0.3 respectively for a pixel to be healthy, symptomatic and background). More generally, in the overall process, we did not get how this classification in three states is used, as probability images rely on continuous measures of probability (see Fig 4C).

Thank you for raising these points that needed to be better explained.

**Action :** We improved the description of those image processing steps to prevent any misunderstanding. (lines 93 – 94, 109 - 112).

Line 295. Why the authors have preferred an anova strategy to compare the estimated parameters on each cultivar (line 82) rather than more classical metrics (eg. AIC with models fitted to all the dataset and considered from 2 to 4 parameters of interest?)

Indeed, using AIC criterion and nested F-test would have be relevant for comparing cultivars. We believe that our approach that consisted in performing ANOVAS on the estimates of parameters is probably easier to understand for biologists whereas it remains appropriate to answer the question. Moreover, F-test with nested models could be influenced by the large number of data we have when using images.

SI3: The figure is labelled as S1 (instead of S3) and refers to Fig. 1c instead of, presumably Fig. 4c

SI5: It in not clear what are the grey vertical lines in the boxplots. Moreover, it would be useful to indicate the range of the number of points (we guess pixels) used to draw each boxplot.

The « grey » lines are actually numerous outliers. The new figures included in the Appendix (now Appendix S3) allow one to visualize the large sample size.

Figure 2 : add a title (and unit) on the x-axis. Moreover, the Jaccard Index is expressed between 0-1, while in the text is presented as a percentage (line 238). Note that at line 74, when talking about Jaccar index, a percentage is missing (it should be 80%).

We thank the reviewer for this comment.

**Action :** We left the Jaccard Index between 0 and 1, deleted % and added the label for the x-axis in the figure.

Additional point: It should be interesting to compare the traits a and D estimated in the present work to the traits classically estimated on this same pathosystem (references 17 and 20). The authors could comment these differences and possibly further argue on the adding value of their framework.

**Action :** We modified the text in the results (lines 227 - 230) and the discussion (lines 256 - 259) to explicit the added value of our results in comparison with previous knowledge. Thank you.
