# Supplementary material for: Imaging with spatio-temporal modelling to characterize the dynamics of plant-pathogen lesions
Source: PLoS Comput Biol. 2023 Nov 20;19(11):e1011627. doi: 10.1371/journal.pcbi.1011627 (PMC10695395; doi:10.1371/journal.pcbi.1011627)
Supplement: Attachment — Submitted filename: Response_to_reviewers_2.docx [file pcbi.1011627.s012.docx]

Dear Editor,

Thank you for your having arranged such thorough and useful reviews of our article “Imaging with spatio-temporal modelling to characterize the dynamics of plant-pathogen lesions” submitted to PLoS Computational Biology. We thank the reviewers for their continued constructive comments that helped us improve the manuscript. We have revised the paper by taking into account all the comments raised by the reviewers. The changes made to the manuscript and the response to the reviewers are detailed below.

Thank you again for considering our article,

Yours sincerely,

Melen Leclerc

On behalf of the co-authors: Stéphane Jumel, Frédéric Hamelin, Rémi Treilhaud, Nicolas Parisey & Youcef Mammeri

The authors did a major effort to answer to our questions and to integrate our comments. In our view this new version of the paper is much more easy to follow and understand, giving some important precisions to the points raised by the reviewers. However, we still have some minor comments.

We are thankful to the reviewers for their careful reading of the manuscript and their relevant comments.

1)We previously showed our concern about the spatial and temporal correlation in pixel data. In the Material and Methods section of the new version of the MS, the authors highlighted that they neglected this correlation. However, contrarily to our suggestion, they did not discuss the implication of this strong assumption. We still believe that at least the nature of the possible bias in parameter estimates (mean value and/or confident interval) generated by this assumption should be mentioned and discussed as a possible extension/improvement of the method. The reader could ask themselves whether these possible biases are negligible with dataset including tens of thousands of pixels. This is why we suggest to more deeply discuss this issue.

Response: We agree that neglecting spatio-temporal correlation of pixel data is a strong hypothesis that is known to bias parameters estimates (mean and variance). However, in our study we believe that the chosen classifier(s) used to produce probability maps might have a stronger impact on parameter estimation.

**Action:** we discussed this issue in the Discussion and added the following sentences: « *For practical reasons we ignored any spatial and temporal correlation in pixel data for parameters estimation. As such a strong assumption can bias parameters estimates (mean and variance), it would be worth considering correlated errors in future works ~\cite{chabot2015}. Yet, for comparing cultivars and isolates, we believe that the bias introduced by the classifier might have a stronger impact on parameters than the assumption of uncorrelated data.* ». As the discussion is already quite long we tried to make it brief.

2)The authors updated the text at lines 91-116 for describing image processing. However, regarding one of our previous comment, it is still not clear why some pixels can be classified as “background” at step 3, after having applied the SIOE algorithm, which already extracted the stipules from the image, at step 1. Is the “background” at line 94 different from the “background” at line 98?

Response: As we stated in the second version of the manuscript the image produced after applying the SIOX algorithm was extracted stipules on a white background rather than a mask. For practical reasons it is easier to train an apply the classifier to these images, the background being obvious and easy to find, rather than on a mask in the initial image.

**Action**: In order to clarify this point, we included the following sentence « *At this step we saved images with stipules on a white background instead of stipules masks (i.e., binary images) »*. We felt that changing the text for step 3 was not necessary and could potentially lead to misunderstanding. We believe that the new sentence prevents any misunderstanding regarding what had been done.

3)Although we get the sense of lines 179-183, we think that the computation of the lesion size from probability maps is not easy to understand. We suggest to explain this point in two steps. Firstly, the authors estimated the observed (respectively estimated) lesion size by counting the number of pixel with probabilities higher than 0.5 from probability maps (respectively model outputs), for each image. Secondly, the authors compare these observed and estimate values in Fig. 4. Another question here is whether the authors considered the 32 images (16 images X 2 cultivars) or the 64 images (16 images X 2 stipules X 2 cultivars)? Another question is whether the lesion size was estimated only at day 7th? Finally, the author could consider to distinguish the points in Fig.4 for James and Solara cultivars with different shapes.

Response: We are thankful to the reviewer for raising this lack of clarity. Symptomatic surfaces were actually estimated on each image used for parameter estimation: 2 cultivars x 4 days x 16 inoculated pairs of stipules.

**Action:** we followed the reviewer’s suggestions. Lines 179-183 now read « *In order to evaluate the ability of the model to capture to lesions sizes rather than probabilities of infection, we estimated symptomatic surfaces for each image by counting the number of pixels with probabilities higher than 0.5 in probability maps and model outputs, and then compared the estimated values*. ». We also included two colours (black and grey) in Figure 4 to distinguish cultivars, and included more details in the caption with the following sentences « For each cultivar, i.e., James (black points) and Solara (gray points), there are thus 16 stipules x 4 dates points. The black line is the first bisector that indicates a perfect agreement between values while the red line is the estimated linear relationship between prediction and observation considering all data (slope $=1.05$, intercept $=-3392.0$). In detail, the relationship for James cultivar was better (slope $=1.01$, intercept $=-1418.7$) than for James and (slope $=1.14$, intercept $=-6947.1$) (relationships not shown in the figure) ».

4)Line 223 the values of coefficient are (likely) inverted. They should be:
“The spread of the pathogen on James cultivar was characterized by mean growth rate a and diffusion coefficient D of respectively 1.33 and 0.55 against 1.26 and 1.54 on the more susceptible Solara.”

Response: Thank, indeed the values were inverted.

**Action**: The values were corrected in the manuscript.

5)At line 224 and Fig.5, the authors should mention (if we understood correctly) that the distributions are based on the 32 values estimated for each cultivar.

Response: As we inoculated 16 pairs of stipules for each cultivar, the distributions are obtained from 16 estimated values. We agree that it is worth mentioning it in the caption.

**Action**: we modified the sentence that is now « *The 32 estimated parameters (16 for each cultivar) are available in \nameref{S8_Appendix}* ».

6)Appendix 3: why not jittering the outliers in figures S32-34? Moreover, the authors should improve figures’ S1-S31 axes in the appendix 3, they are really difficult to read.

**Action**: we followed the advice and deleted outliers in the boxplots and increased the size of figures S1-31 so one can easier read the axes and figures.

In addition to our previous comments, we would like to raise few additional points:

1)For the readers of PLoS CB, it could be interesting that the authors shortly explain why “it is more relevant to rely on data assimilation method (line 151-152)”.

Response: Data assimilation is a broader framework that includes parameter estimation. In our case the variational approach had the advantage of improving the numerical resolution of the optimization problem that included numerous data as we considered image pixels.

**Action**: We modified lines 151-152, that now read « *When observations are image sequences, parameter estimation can be performed using some data assimilation methods used to fit models to image data, for instance in fluid dynamics~\cite{papadakis2008} or biomedical modelling~\cite{mang2020}.* » and included a new sentence in lines 159-160 to explain the advantage of the variational approach : « *This approach enables a more efficient numerical optimization and thus a faster parameter estimation* ».

2)To avoid repetitions from the Image processing section, the authors could consider to rephrase lines 159-163 with only: “In our cases the probability images (Figure 1) were considered as the observation ureg(x,t ) in the cost function.”

Response: Since we discuss this point in the Discussion we agree that this section could be shortened.

**Action**: we rephrased lines 159-163 following the reviewer’s suggestion.

3)Figure 2: “stipules of Solara (a) AND (not ET) James (b)”. Also, the authors should mention in the legend that “1” and “17” are plant numbers.

Response: Thank you for highlighting this typo, and for the suggestion.

**Action**: We corrected the typo and included the information regarding the stipule numbers in the legend.

4)At line 120: how is the fit quality of non-spatial model?

Response: Goodness-of-fit tests based on Residual sum of squares supported the hypothesis of good fits (e.g., Leclerc et al. 2019). However, as these works did not consider spatial data and considered the global lesion size, such analyses also hid various discrepancies.

5)At line 125, replace with “appearance of symptomatic host tissue” ? (as the model does not consider leaf growth).

Response: As we focus on the spread of lesions, we agree that it makes sense to rephrase as suggested by the reviewer. However, to prevent any misunderstanding relative to the term“appearance”, here we mean how it looks, and not the appearance of new tissues. Your suggestion will help to prevent such misunderstanding.

**Action**: We included « symptomatic » in the main text (line 125).

6)At line 53, 117 (and in many other parts throughout the paper), the authors state that the model describes the spread of necrotic lesion on host tissue, which is effectively the case. We wonder whether defining u as the “probability that a host is infected” (at line 131), is not somewhat misleading. The author could say that u is the probability of a host at location x at time t to be necrotic as the result of the final step infection process.

Response: Thank you for this interesting comment. As *P. pinodes* is a necrotrophic pathogen it « kills » host cells before the infection. Thus, in this particular case the probability of infection should similar to the probability of being necrotic.

**Action**: We included the sentence « As {\it P. pinodes} is a necrotrophic pathogen we can assume that the probability of being necrotic is similar to the probability of being infected. » in line 131.
